# Global quantitative TPA-based proteomics of mouse brain structures reveals significant alterations in expression of proteins involved in neuronal plasticity during aging

**DOI:** 10.18632/aging.101501

**Published:** 2018-07-19

**Authors:** Przemysław Duda, Olga Wójcicka, Jacek R. Wiśniewski, Dariusz Rakus

**Affiliations:** 1 Department of Molecular Physiology and Neurobiology, University of Wroclaw, Wroclaw 50-137, Poland; 2 Department of Proteomics and Signal Transduction, Max-Planck-Institute of Biochemistry, Martinsried 82152, Germany

**Keywords:** AMPA, GABA, NMDA, long-term potentiation, memory formation

## Abstract

Aging is believed to be the result of alterations of protein expression and accumulation of changes in biomolecules. Although there are numerous reports demonstrating changes in protein expression in brain during aging, only few of them describe global changes at the protein level. Here, we present the deepest quantitative proteomic analysis of three brain regions, hippocampus, cortex and cerebellum, in mice aged 1 or 12 months, using the total protein approach technique. In all the brain regions, both in young and middle-aged animals, we quantitatively measured over 5,200 proteins. We found that although the total protein expression in middle-aged brain structures is practically unaffected by aging, there are significant differences between young and middle-aged mice in the expression of some receptors and signaling cascade proteins proven to be significant for learning and memory formation. Our analysis demonstrates that the hippocampus is the most variable structure during natural aging and that the first symptoms of weakening of neuronal plasticity may be observed on protein level in middle-aged animals.

## Introduction

Aging is associated with general decline in cognitive functions [1]. It is widely accepted that aging affects brain plasticity via changes in mechanisms regulating synaptic plasticity like long-term potentiation (LTP) and long-term depression (LTD) [2,3]. However, the molecular mechanisms of such decline in cognitive abilities of older individuals remain elusive.

The effects of aging on the level of proteins and gene expression in the hypothalamus, cortex and cerebellum of rodents has been investigated by microarrays [4–6] and by mass spectrometry-based proteomic techniques [7–9]. Except Walther and Mann’s study [7], all the proteomic studies on brain aging have been performed using semi-quantitative techniques (usually based on 2D PAGE protein separation and further MS analysis of dissected spots) which do not deliver information about accurate concentration of a protein in studied samples.

The technique used by Walther and Mann to study differences between young adult (5 months) and old (26 months) rats has revealed a relative stability of brain structures proteomes during aging [7]. More recent proteomic studies on age-associated changes in various brain structures, e.g. on aging of human and mice hippocampus [10,11], are roughly in line with the results of Walther and Mann, however, they have demonstrated significant changes in titers of enzymes catalyzing regulatory reactions in energy metabolism which is reflected by basically different organization of astrocyte-neuron crosstalk in young and old hippocampus [11]. One of the most recent quantitative study on aging of human brain structures [12] has presented data from a very small group which makes the data hard to interpret.

In this study, we provide the deepest quantitative descriptions of mice hippocampal, cerebellar and cortical proteomes during normal/physiological aging. Studying the effect of aging we identified more than 8,500 proteins and we measured quantitatively 5,288, 5,679 and 5,757 proteins in, respectively, hippocampus, cortex and cerebellum in 1 month- and 12 month-old mice. Our analysis demonstrates that aging does not affect significantly the total abundance of proteins in the studied brain structures, however, the levels of many individual proteins differs significantly. Unexpectedly, the highest amount of proteins which expression was significantly altered by aging was observed in hippocampus, while cortex was the most stable structure.

In our analysis, we focused on the changes in the expression of synaptic proteins involved in neuronal plasticity. The results presented here demonstrate that the first proteomic symptoms of weakening of signal transmission and neuroplasticity may be observed in the middle-aged (12 month-old) animals. A detailed discussion of a meaning of these changes is given below.

## RESULTS AND DISCUSSION

### Proteomic analysis

**Figure 1:**
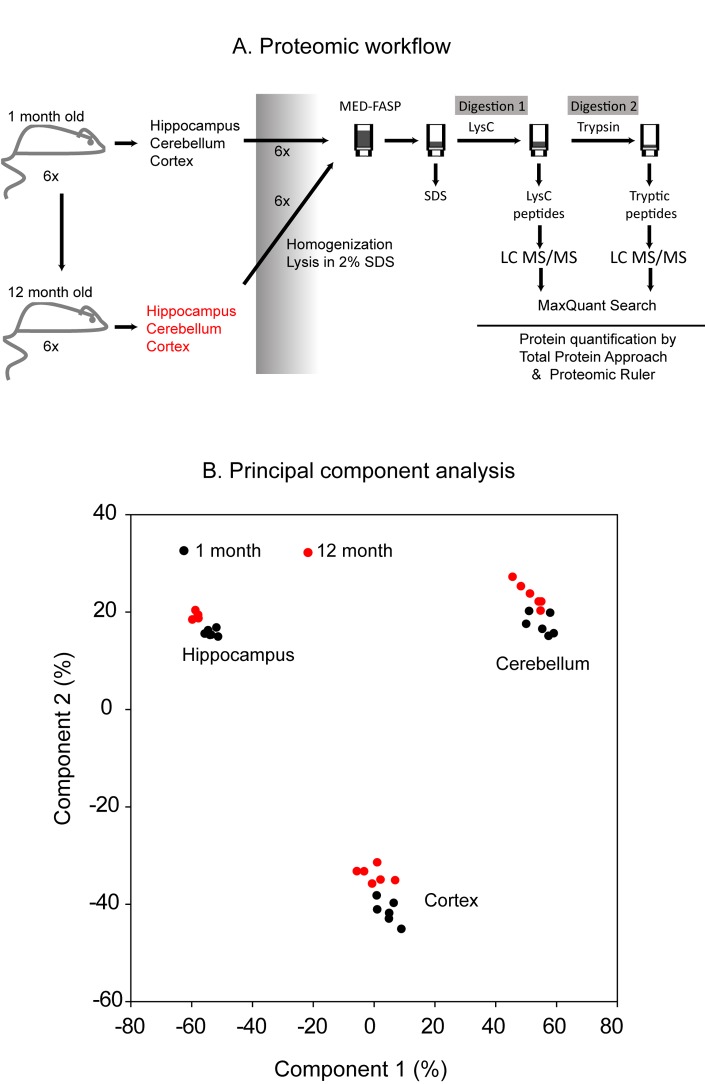
**Proteomic workflow.** Cerebellum, cortex and hippocampus were isolated from 1 month- and 1 year-old mice. Six animals were used to analysis at each age. The tissues were homogenized and lysed in a buffer containing 2% SDS. The lysates were processed with the multiple enzyme digestion filter aided sample preparation (MED FASP) method using consecutive cleavage with LysC and trypsin. The protein digests were analyzed by LC-MS/MS and the spectra were processed with MaxQuant software. Specific protein concentrations (mol/g total protein) were obtained by the ‘Total Protein Approach’ and protein copy numbers were assessed using the ‘Proteomic Ruler’ method (**A**). Principal component analysis based on the protein concentration values. Only proteins with at least 2 peptides were considered (**B**).

Cerebellum, cortex and hippocampus were dissected from 1 month- and 12 month-old mice (Figure 1A). The tissues from each mouse were analyzed separately by global proteomics. The sample preparation procedure involved solubilization of proteins in a buffer containing 2% SDS [13] and a two-step consecutive proteins digestion by MED FASP approach using LysC and trypsin. Aliquots of peptides obtained by each cleavage were separately analyzed by LC-MS/MS. The analysis of the spectra allowed identification of 8,507 proteins across the analyzed samples (PRIDE partner repository, the dataset identifier: PXD009792). For quantitative analysis, only proteins with at least 2 unique peptides were used (7732 proteins in total). Principal component analysis of protein concentrations revealed age-related changes in the composition of proteomes in each of the analyzed tissue (Figure 1B). Notably, as expected, huge proteome differences were observed between cerebellum, cortex and hippocampus.

### The effect of aging on total protein expression in brain structures

The expression data for about 5,700 proteins in cortex and cerebellum, and for almost 5,300 proteins in hippocampus of each of the four young and middle-aged mice was quantitatively determined and deposited to the ProteomeXchange Consortium via the PRIDE partner repository [14] with the dataset identifier: PXD009792 (for the review: username: reviewer16980@ebi.ac.uk and password: FpqJ9qms).

**Figure 2:**
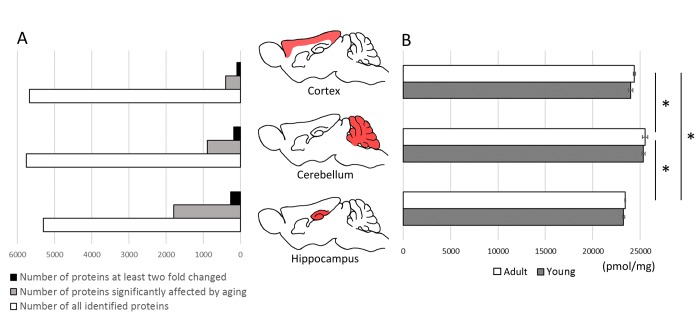
**The effect of aging on total protein expression in brain structures.** The comparison of proteins identified quantitatively in cortex, cerebellum and hippocampus and the number of proteins affected significantly (p<0.05) by aging (**A**).The total molar (pmol/mg) concentration of proteins in brain formations of young and middle-aged mice (**B**). Asterisks indicate a statistically significant difference (p < 0.05).

Total molar protein concentration was statistically the highest in cerebellum, both in young and in middle-aged animals, while the lowest was in hippocampus (Figure 2B). The differences between the proteomes of individual animals in total protein concentration were negligible and were below 1%.

Aging had no effect on the total titer of proteins, however, the concentration of several individual proteins was significantly altered.

The highest number of changes was observed in hippocampus where more than 1760 proteins were affected by aging (p<0.05) (Figure 2A). In cortex and cerebellum, the changes (p<0.05) were attributed to, respectively, 391 and 889 proteins (Figure 2A). Among these proteins, the titer of 256, 97 and 219 proteins, respectively, in hippocampus, cortex and cerebellum was changed at least two fold (Figure 2A, Table S1). In all studied brain formations, the concentration of about two-third of the proteins was significantly decreased in middle-aged animals (Table S1).

### Glutamate receptors

Glutamate is the main excitatory neurotransmitter in brain which exerts its action through receptors that function as ion channels, such as AMPA receptors (which are heterotetrameric proteins containing Gria subunits), NMDA receptors (formed by Grin subunits), kainate receptors (containing Grik isoforms) and Grid receptors, and also via signaling cascades associated with metabotropic receptors (Grm, also known as mGlur).

### *AMPA receptors*

**Figure 3:**
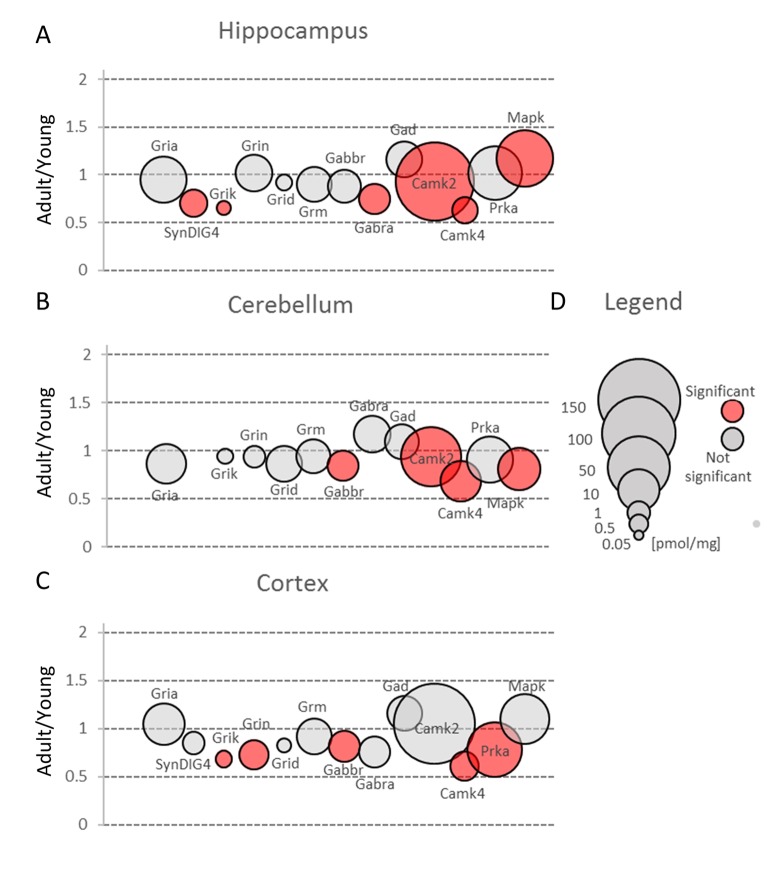
**Changes in protein families involved in main mechanisms of neuronal plasticity upon aging.** Plots show ratios of protein concentrations in adult vs young brain structures: hippocampus (**A**), cerebellum (**B**) and cortex (**C**). The size of bubbles is proportional to the average protein concentration in respective young animals brain structures. Statistically significant differences are shown as the red filled circles.

AMPAR (Gria) are the most abundant ionotropic glutamate receptors in hippocampus, cortex and cerebellum (Figure 3, Table S2). They are the main type of glutamate receptors that mediate fast excitatory synaptic transmission and are crucial for the expression of various forms of long-lasting synaptic plasticity including long-term potentiation (LTP) and long-term depression (LTD) [15]. Thus, it is not unexpected that we found the highest concentration of Gria proteins in hippocampus, the structure in which new memories are formed [16] (Figure 3A, Table S2).

**Figure 4:**
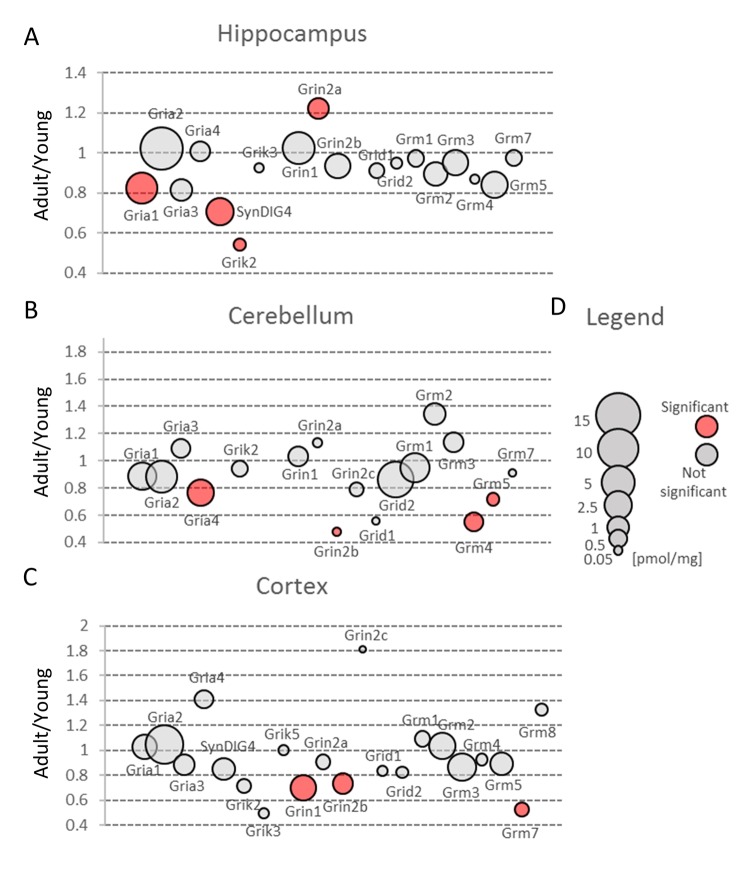
**Changes in expression of proteins involved in glutamatergic transmission upon aging.** Plots show ratios of protein concentrations in adult vs young brain structures: hippocampus (**A**), cerebellum (**B**) and cortex (**C**). The size of bubbles is proportional to the average protein concentration in respective young animals brain structures. Statistically significant differences are shown as the red filled circles.

In all brain structures, the most abundant isoform was Gria2, while Gria1 was the second most ubiquitous isoform (Figure 4, Table S3). The concentration of Gria3 and Gria4 was much lower, however, in cerebellum, Gria4 subunit was expressed at a similar level to Gria1 (Figure 4B, Table S3). This is consistent to a previous study showing that Gria4 is one of the main isoform in cerebellum [17], however, in contrast to that report, we did not see a high expression of Gria1 in this region of brain. The role of Gria4 is poorly understood. It has been hypothesized that this isoform may play a role in delivery of Gria2-containing AMPA receptors into synapses in postnatal hippocampus [18]. It has been also suggested that Gria4 may be involved in working and reference memories, however, the mechanism is elusive [19].

**Figure 5:**
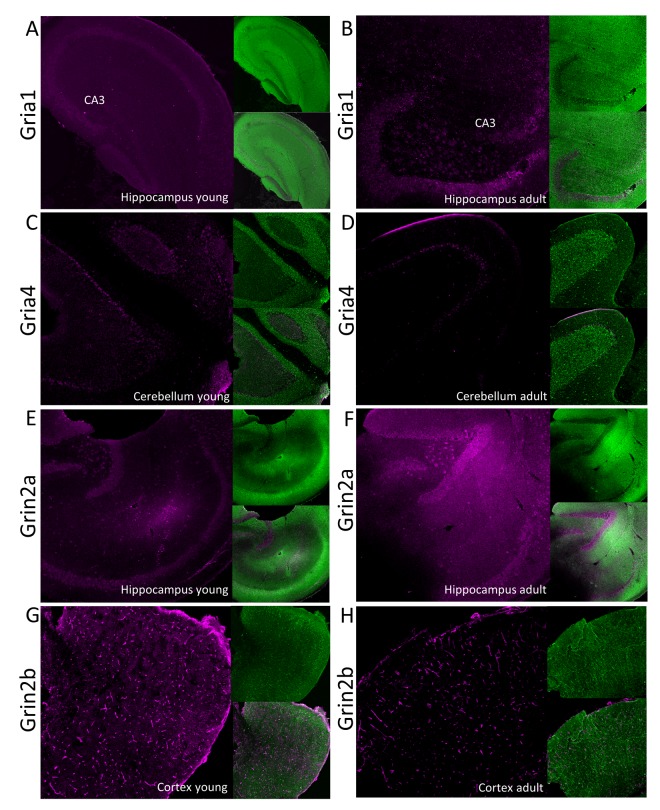
**Immunofluorescent localization of selected glutamate ionotropic receptors which expression is altered by aging.** Panels **A-D** show expression of AMPA receptor subunits: Gria1 (**A** and **B**) and Gria4 (**C** and **D**) in, respectively, hippocampus and cerebellum. Panels **E-H** show fluorescent signal associated with NMDA receptor subunits: Grin2a (**E** and **F**) and Grin2b (**G** and **H**) in, respectively, hippocampus and cortex. Panels **A**, **C**, **E** and **G** show the receptors localization in young, while the right panels (**B**, **D**, **F** and **H**) in aged animals. In each panel, the fluorescence related to studied proteins is shown in magenta (left images), the signal associated with MAP-2 protein is green (right upper image) and the merged image is presented in the left bottom image. CA3 – Cornu Ammonis area of hippocampus containing pyramidal neurons.

Our proteomic analysis showed that a total concentration of Gria proteins was unaffected by aging. However, we found a statistically significant (about 20%) decrease in Gria1 titer in aged hippocampus (Figure 4A, Table S3) and Gria4 in aged cerebellum (Figure 4B, Table S3). Interestingly, although the changes were below 25%, it was possible to see differences using the immunofluorescent methods (Figure 5A and B, Figure 5C and D). Especially evident changes were detected in hippocampus, where the decrease in Gria1 was accompanied by age-associated disruption of CA3 area integrity (Figure 5A and B). It has been shown that the absence of Gria1 in AMPAR was associated with deficit in spatial working memory [20] and that aging-associated cognitive decline correlated with reduced amount of Gria1 on the surface of postsynaptic membrane in 22 month-old rats as compared to 6 month-old animals [21]. In the latter study [21], the authors have found, using a semi-quantitative method (Western Blot analysis), that a cell surface expression and phosphorylation of Gria1 (GluR1) in the hippocampus of middle-aged rats has been significantly decreased although the total abundance of Gria1 has been unaffected by aging. The quantitative data presented here demonstrated that total Gria1 titer decreased significantly in hippocampi of one year-old mice (Figure 4A, Table S3).

Interestingly, Gria1 recruitment to postsynaptic density after LTP induction has been shown to be regulated by lateral diffusion [15] in which SynDIG4 protein is supposed to play the crucial role [22]. It has been demonstrated that SynDIG4 co-localizes with Gria1 subunit of AMPAR at extrasynaptic region and it maintains a pool of the receptors needed for synaptic plasticity [22]. Results presented here revealed that the titer of SynDIG4/Prrt1 was significantly, about 30%, reduced in hippocampi of middle-aged mice as compared to the young ones (Figure 4A, Table S3). Together, the observed changes in Gria1-SynDig4/Prrt1 expression suggest that just a decline in Gria1 and SynDIG4 may be the main cause of a decreased plasticity during aging.

### *NMDA receptors*

In the classic mechanism of LTP induction, AMPA receptors are recruited to postsynaptic region after NMDA activation. Our analysis did not show significant differences in total expression of NMDA receptor subunits (Grin) in hippocampus and cerebellum (Figure 3A and B, Table S2), while in cortex the total molar concentration of Grin was significantly, almost 30%, reduced (Figure 3C, Table S2). As in the case of AMPA receptors, the highest expression of Grin was attributed to hippocampal formation.

The only age-related statistically significant differences observed in hippocampus were associated with slight elevation of Grin2a (Figure 4A, Table S3) which was reflected by a higher immunofluorescent signal coming from anti-Grin2a antibodies (Figure 5E and F).

Interestingly, NMDA receptors containing Grin2a subunits express a synaptic localization and they have faster kinetics than channels formed by Grin2b subunits which titer was not affected in hippocampi of aged mice (Figure 4A, Table S3). In contrast to hippocampus, concentration of Grin2b was significantly reduced in aged cerebellum in which the level of Grin2b was more than two times lower in middle-aged mice, and in cortex (Figure 4B and C, Table S3) where the proteomic changes were reflected by Grin2b fluorescent signal (Figure 5G and H). In contrast to Grin2a, Grin2b isoforms have high affinity to glutamate and are located mainly extrasynaptically [23]. Both proteins have also different co-agonists: D-serine for synaptic NMDA channels and glycine for extrasynaptic receptors [23]. In contrast to Grin2a, the Grin2b receptor participates predominantly in long-term depression (LTD). Interestingly, it has been shown that Grin2b stimulation occurs mainly during ischemic episodes when glutamate is released from glia and neuronal cells via vesicle-independent mechanism and as a result of this, massive opening of extrasynaptic NMDA receptors containing Grin2b induces neuronal damage [23]. Thus, it might be hypothesized that reduced amount of “apoptotic” Grin2b subunits is a mechanism protecting aging cortex and cerebellum from the loss of neurons.

### *Grid, kainite and metabotropic glutamate receptors*

We did not observe significant aging-related changes in Grid ionotropic receptors which were the least abundant receptors for glutamate in hippocampus and cortex but the second most highly expressed glutamatergic receptors in cerebellum (Figure 3, Table S2).

The kainate family of glutamate receptors (Grik) was predominantly expressed in cortex (Figure 3C, Table S2). The expression of proteins forming kainite receptors did not show any statistically significant age-dependent alterations in the cerebellum, however, we observed their statistically significant (about 1.5-fold) decrease in hippocampus and cortex (Figure 3A and C, Table S2). The largest statistically significant (almost 2-fold) decrease concerned Grik2 level in hippocampus (Figure 4A). Grik proteins are located both pre- and postsynaptically and they have been shown to modulate an excitatory and inhibitory transmission and to affect short- and long-term memory [24].

Mice lacking Grik2 (also known as GluR6) exhibit the impairment in short-term synaptic facilitation and the reduced LTP in mossy fibers [25]. Thus, the decline in Grik2 expression may contribute to aging-associated decrease in hippocampal plasticity in middle-aged individuals.

Metabotropic glutamate receptors (Grm, mGlu) belong to G-protein-bound receptors acting via secondary messengers like cyclic AMP, inositol 3-phosphate and Ca^2+^.

Our proteomic analysis showed that in all studied brain structures, Grm proteins were present at similar level and aging did not affect significantly their total expression (Figure3, Table S2). On the other hand, our analysis of cerebella of healthy middle-aged mice revealed a significant decrease in concentration of Grm4 and Grm5 (Figure 4, Table S3). The role of Grm5 in cerebellum is not fully understood but in hippocampi, the activation of Grm5 have been shown to facilitate transformation of short-term potentiation and short-term depression into their persistent forms, respectively, LTP and LTD [26]. There is also convincing evidence that Grm5, together with kainite receptors, is involved in the mechanism of anti-Hebbian (AMPA/NMDA-independent) LTP formation in mossy fibers [27]. Moreover, it has been shown that activation of Grm1 and Grm5 is required for induction of anti-Hebbian LTP in hippocampal interneurons [28] and that the disruption of Grm5 signaling is associated with several disorders such as autism, depression and movement disorders [29–31]. However, the animals used in our studies demonstrated no hallmarks of neurological disorders. Thus, it might be speculated that the role of Grm5 in development of neuropathological states may be overestimated.

### Gamma-aminobutyric acid receptors (GABAR)

GABA is the primary inhibitory neurotransmitter of the central nervous system [32].

Receptors for GABA (Gabr) are divided into two classes: the ionotropic Gabra is a chloride channel, while the metabotropic (Gabbr) action is usually associated with membrane hyperpolarization caused by opening of potassium channels and/or inhibition of calcium channels [32].

In our study, we observed substantial changes in both Gabra and Gabbr concentration during aging (Figure 3, Table S2).

**Figure 6:**
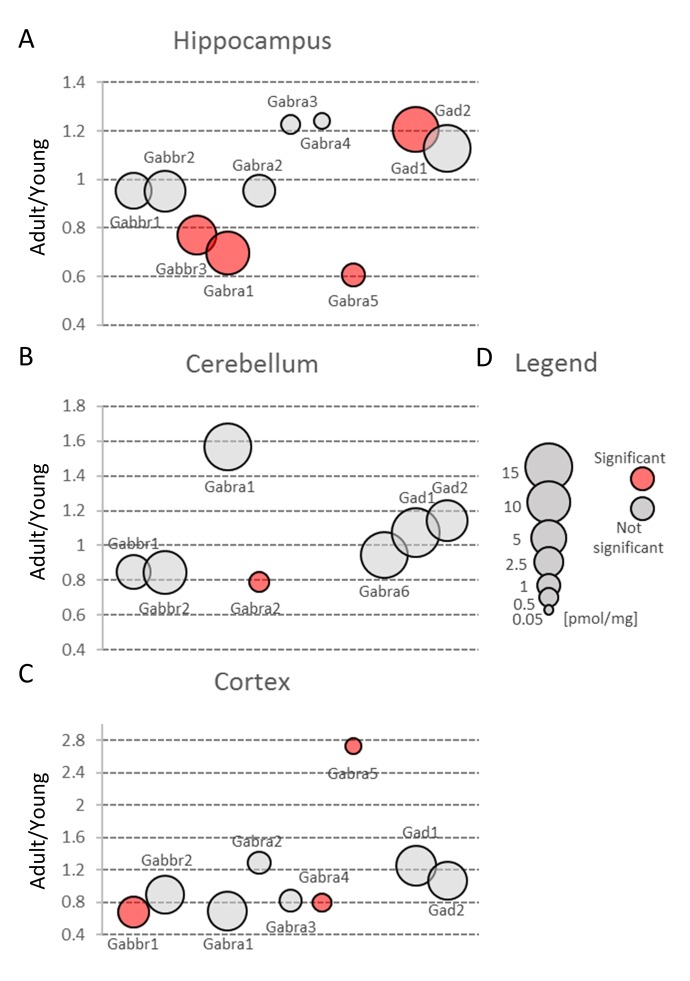
**Aging-related changes in proteins involved in GABAergic transmission.** Plots show ratios of protein concentrations in adult vs young brain structures: hippocampus (**A**), cerebellum (**B**) and cortex (**C**). The size of bubbles is proportional to the average protein concentration in respective young animals brain structures. Statistically significant differences are shown as the red filled circles.

Our proteomic analysis revealed that concentration of Gabra was significantly decreased in the middle-aged hippocampus (Figure 3, Table S2). The expression of some members of Gabra family, Gabra4 and Gabra5, was also altered in the cortex (Figure 6C) but because these isoforms are produced at very low level their impact on total GABA receptors production seems to be negligible. Hippocampal changes were attributed to Gabra1, the most abundant isoform of the receptor (Figure 6A, Table S3). Our results on the expression of Gabra confirm earlier electrophysiological studies which demonstrated a reduced inhibitory transmission in the hippocampi of aged animals [32].

Small changes in Gabbr proteins level were observed in all studied brain structures, but a statistically significant decrease in Gabbr were associated with cortex and cerebellum (Figure 3B and C, Table S2).

In contrast to previous studies [33], we did not observe any reduction in Gad protein in all studied brain structures (Figure 3, Table S2). Gad is an enzyme producing GABA via glutamate decarboxylation and it has been hypothesized that a decreased inhibitory transmission mediated by GABA contributes to memory impairment in 15 month-old rats [33] and spatial memory deficits in 26 month-old rats [34]. Our data does not exclude that in old animals, a decrease in Gad level may be observed, however, the changes in middle-aged animal brains, especially in hippocampi, suggest rather compensatory increase in Gad as a response to a decreased abundance of GABA ionotropic receptors (Figure 6A, Table S3).

### Kinases involved in memory formation: Camk, Prka and Mapk

### *Camk2*

**Figure 7:**
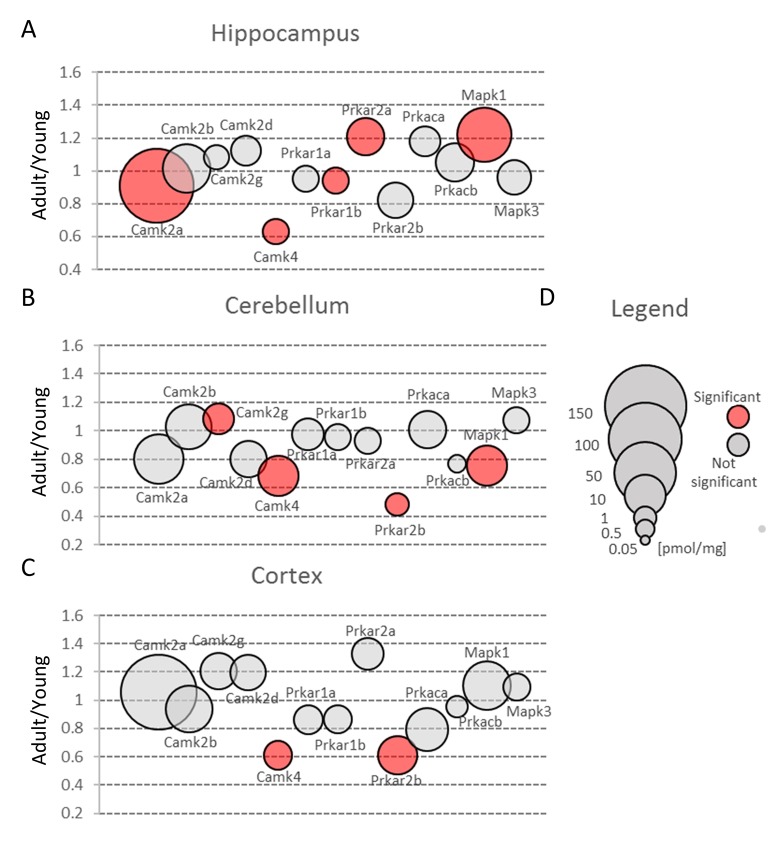
**Protein kinases involved in glutamate-induced neuronal plasticity.** Plots show ratios of protein concentrations in adult vs young brain structures: hippocampus (**A**), cerebellum (**B**) and cortex (**C**). The size of bubbles is proportional to the average protein concentration in respective young animals brain structures. Statistically significant differences are shown as the red filled circles.

Most of the plasticity phenomena, like LTP and LTD, are initiated by the entrance of calcium ions into postsynaptic bulb via NMDA or AMPA receptors, and the activation of calcium/calmodulin-dependent protein kinase (Camk) is the first stage of transformation of calcium signaling into various form of plasticity [35]. Camks are ubiquitous proteins in all brain structures and it has been suggested that they may comprise up to 2% of the total hippocampal proteins and about 1.3% of cortical proteins [36]. Our study confirmed that Camk is abundantly expressed in all studied brain formations (Figure 3, Table S2). However, we found that its participation in the total amount of protein is much lower than it had been reported earlier [36]. We found that Camk2, which is the predominant form of Camks (Figure 7, Table S3), made up about 0.6% of hippocampal proteins, 0.55% of cortical and about 0.2% of cerebellar proteins.

Nonetheless, the amount of Camk2 isoforms was many-fold higher than the titer of other protein kinases and similar to the abundance of basic metabolic enzymes such as aldolase and pyruvate kinase. Such extremely high expression of the protein kinase raises the question of whether the role of Camk2 (especially it’s the most abundant isoform, Camk2a) goes beyond its catalytic function. It has been shown that Camk2a may interact with several proteins, e.g. Grin2b [37] and Camk2b, the second most abundant Camk2 isoform, may associate with fibrillar actin and regulate actin filaments stability [38] which, in turn, regulates the organization of the postsynaptic regions in glutamatergic synapses [39]. Our proteomic analysis revealed that the total Camk2 amount in hippocampus and cerebellum was statistically significantly decreased by aging (Figure 3A and B, Table S2). Although the changes were not very large, they suggest that decrease in Camk2 may affect the proper structural organization of postsynaptic region and in this manner, may be involved in age-related changes in excitatory transmission.

### *Camk4*

Camk4 is a member of Camk family but its molecular role in LTP is different than Camk2. Active Camk4 localizes in cell nucleus and takes part in late phase of LTP formation [40] by regulating gene transcription via activation of cyclic AMP responsive element binding protein (CREB) [41].

The kinase has been shown to localize in various structures of brain, mainly in cerebellum, cortex and hippocampus, and results of our proteomic study are roughly in line with the earlier histochemical studies [42]. An unexpected observation was that the amount of Camk4 in cerebellum was many times higher than in the other two studied structures (Figure 3B, Table S2).

Our results also demonstrated that the amount of Camk4 strongly decreased in all studied structures during aging (Figure 7, Table S2) which is in line with the previous studies showing that Camk4 expression in hippocampus declines with aging [43] and which suggests that, even in middle-aged animals, the ability to form a new memory is much weaker than in young ones.

### *Prka*

In contrast to Camk4, the total amount of Prka (PKA) subunits, the second kinase crucial for stimulation of gene transcription during induction and formation of various forms of memory [44], was unaffected by aging in hippocampus and cerebellum (Figure 3A and B, Table S2). On the other hand, we observed a statistically significant decrease of Prka in cortex (Figure 3C) but the changes were attributed only to a regulatory subunit, Prkar2b (Figure 7C, Table S3). Small but statistically significant changes were also attributed to regulatory subunits in hippocampus (Prkar1b and Prkar2a) and cerebellum (Prkar2b) but they did not affect the total amount of Prka (Figure 3A and B, Figure 7A and B).

PKA exhibits low substrate specificity and it phosphorylates several proteins both in nucleus and in cytoplasm. It can phosphorylate e.g. various subunits of AMPA and NMDA receptors and modulate their function [45]. Thus, the observed changes in the regulatory subunits expression may reflect the first symptoms of age-associated downregulation of memory formation mechanisms.

### *Mapk*

Long-term neuronal plasticity is associated with changes in the level of transcripts for multiple genes. During the last years it has been demonstrated that several molecular cascades, except the canonical one mediated by NMDA-Camk2-Camk4, may lead to persistent changes in strength of synaptic connections [45].

The extracellular signal-regulated kinase (Erk) belongs to the mitogen-activated protein kinases (Mapk), a superfamily of proteins which members regulate a broad spectrum of cytoplasmic and nuclear processes in neuronal plasticity [46,47]. Erk activation by variety of intracellular pathways/factors, like an elevated calcium level and Ras activation, has been shown to be involved in the formation of LTP via regulation of the activity of transcription factors [47].

Our analysis demonstrated that the total Erk expression was relatively stable in cortex but it was significantly reduced in cerebellum of middle-aged mice (Figure 3B, Table S2). This resulted from a significant decrease in Erk2 (Mapk1) level (Figure 7B). In contrast to cerebellum, the amount of Erk2 in hippocampus was statistically significantly elevated (Figure 7A, Table S3) and, as Erk2 is the predominant isoform in brain, it was reflected by slight increase in total abundance of Erk isoforms in this formation (Figure 3A).

Interestingly, we found that in hippocampus and cortex, the amount of Erk isoforms (notably Erk2) was many times higher than Camk4 which is regarded to be crucial for long-term synaptic plasticity formation via phosphorylation of transcription factors (Figure 7). We also found that, in contrast to Camk4, the level of Erk was the highest in hippocampus and the lowest in cerebellum (Figure 3) what supports previous suggestion that Erk-associated pathway may be the major route for CREB activation (and memory formation) in hippocampus [48].

### Concluding remarks

In this manuscript, we deliver the deepest quantitative description of hippocampal, cortical and cerebellar proteomes of young and middle-aged mice.

Our analysis demonstrates that hippocampus is the most variable structure during natural aging and that in middle-aged animals, the machinery involved in the formation of various forms of hippocampal neuronal plasticity is significantly altered.

We found that although the total protein expression in middle-aged brain structures is practically unaffected by aging, there are significant differences between young and middle-aged mice in the expression of some receptors and signaling cascade proteins which significance for learning and memory formation has been demonstrated.

The synchronous decrease, especially in hippocampal formation, in the expression of proteins crucial for brain plasticity but belonging to various protein families and participating in different forms of learning and memory formation (e.g.: Gria1, SynDig4/Prrt1, Grik2 or Camk4) suggests that a common mechanism may underlie the observed age-associated alterations. Just recently, we have shown that aging is accompanied with reorganization of hippocampal energy metabolism which is manifested by elevated capacity of aging neurons to oxidize glucose in glycolysis and in mitochondria, and decreased ability for fatty acids utilization [11] and disruption of LTP formation [49]. Lately, it has been also suggested that aging-related changes in glycolysis may lead to a decrease in synaptic plasticity and development of Alzheimer’s disease [50]. Thus, an attractive hypothesis is that the changes in neuronal energetic metabolism may be a trigger switching off and/or dysregulating the expression of proteins involved in brain plasticity. Consequently, manipulation of brain energy metabolism (and astrocyte-neuron crosstalk) may be a promising method for reversal of age-related changes in neurotransmission.

## MATERIALS AND METHODS

### Animals and tissue preparation

The experiments were performed on 2 groups of male C57BL/10J mice: young (P30, n=6) and aged (12 months, n=6). Animals were anesthetized with isoflurane and decapitated. Brain regions were isolated in ice-cold buffer containing (in mM): NaCl 87, KCl 2.5, NaH_2_PO_4_ 1.25, NaHCO_3_ 25, CaCl_2_ 0.5, MgSO_4_ 7, glucose 25, sucrose 75; pH 7.4. The left part of hippocampi, cortex and cerebellum from each animal were analyzed using quantitative proteomics, the right ones were used for immunofluorescence. All the procedures were approved by the local Ethical Commission and every effort was made to minimize the number of animals used for the experiments.

### Preparation of tissue lysates

Brain structures were homogenized immediately after isolation in buffer containing 0.1 M Tris/HCl, 2% SDS, 50 mM DTT, pH 8.0 and incubated 5 min at 99^o^C. Samples were cooled in liquid nitrogen and stored in -20^o^C until proteomic analysis. Total protein was determined by measuring tryptophan fluorescence as described previously [51].

### Multi-enzyme digestion filter aided sample preparation (MED FASP)

Sample aliquots containing 70 μg total protein were processed using the MED FASP method [52] with modifications described recently [53]. Proteins were first cleaved overnight LysC, and in the second step digested with trypsin for 2h. The enzyme to protein ration was 1:50. Digestions were carried out in 50 mM Tris-HCl, pH 8.5 at 37°C. Aliquots containing 10 µg total peptide were concentrated to a volume of ~5 µL and were stored frozen at -20 C until mass spectrometric analysis.

### Liquid chromatography – tandem mass spectrometry

Analysis of peptide mixtures was performed using a QExactive HF mass spectrometer (Thermo-Fisher Scientific, Palo Alto). Aliquots containing 2.5 μg total peptide were chromatographed on a 50 cm column with 75 µm inner diameter packed C_18_ material (100 Å pore size; Dr. Maisch GmbH, Ammerbuch-Entringen, Germany). Peptide separation was carried out at 300 nL/min for 75 min using a two-step acetonitrile gradient 5-40% over the first 85 min and 40-95% for the following 15 min. The temperature of the column oven was 55°C.

The mass spectrometer operated in data-dependent mode with survey scans acquired at a resolution of 50 000 at m/z 400 (transient time 256 ms). Up to the top 15 most abundant isotope patterns with charge ≥ +2 from the survey scan (300-1650 m/z) were selected with an isolation window of 1.6 m/z and fragmented by HCD with normalized collision energies of 25. The maximum ion injection times for the survey scan and the MS/MS scans were 20 and 60 ms, respectively. The ion target value for MS1 and MS2 scan modes was set to 3×10^6^ and 10^5^, respectively. The dynamic exclusion was 25 s and 10 ppm. The mass spectrometry data have been deposited to the ProteomeXchange Consortium via the PRIDE partner repository [14] with the dataset identifier: PXD005230 (for the review: username: reviewer16980@ebi.ac.uk and password: FpqJ9qms).

### Proteomic data analysis

The spectra were searched using MaxQuant software. All raw files were searched together in single MaxQuant A maximum of 2 missed cleavages was allowed. Carbamidomethylation was set as fixed modification. The “matching between the runs“-option was used. The maximum false peptide and protein discovery rate was specified as 0.01. Protein abundances were calculated using the “total protein approach” (TPA) method [54,55]. The calculations were performed in Microsoft Excel using the relationship:

where

$$c\left(i\right)=\frac{MS-signal\;\left(i\right)}{Total\;MS-signal\;\times MW\left(i\right)}\;\left[\frac{mol}{g\;total\;protein}\right]$$

### Immunofluorescence

All the brain parts were fixed in 4% paraformaldehyde immediately after dissection. After 24 hours they were washed 3 times with phosphate buffer saline (PBS), immersed in 2% agarose and cut into 75 µm slices on Tissue Chopper (Ted Pella, USA). The slices were washed in PBS to remove the agarose and 2 times in PBS with 0.1% Triton X100 for cell membrane permeabilization. Next, the primary antibodies were added in an appropriate dilution: anti-MAP- 2 antibody (1:500; Sigma, cat. no. M4403); anti-NMDAR2A antibody (1:100; Sigma, cat. no.SAB4501304); anti-NMDAR2B antibody (1:100; Sigma, cat. no. SAB4501305); anti-GluR1 antibody (1:100; Sigma, cat. no. SAB4501293); anti-GluR4 antibody (1:300; Sigma, cat. no. SAB4501296). The slices were incubated with the primary antibodies for 48h at 4^o^C, washed 3 times with PBS and the secondary antibodies were added: AlexaFluor488 and AlexaFluor633 (1:1000; ThermoFisher Scientific, Molecular Probes, USA). The slices were incubated with the secondary antibodies for 2h at room temperature. Next, slices were washed with PBS and immersed in Fluoroshield (Sigma, F6182).

Tissue sections were examined with the FV1000 confocal microscope (Olympus, Tokyo, Japan) using 10x objective and scanned at 2,048 x 2,048 picture resolution.

### Statistical analysis

All results are presented as mean ± *SEM* unless otherwise stated. The statistical analysis was performed using Student’s t test preceded by Fisher F test. We used non-paired Student’s t test for comparisons between any two experimental groups. The statistical analyses were performed using SigmaPlot 11 software (Systat Software).

## Supplementary Material

Supplementary Table S1

Supplementary Table S2

Supplementary Table S3
