# Supplementary material for: Global quantitative TPA-based proteomics of mouse brain structures reveals significant alterations in expression of proteins involved in neuronal plasticity during aging
Source: Aging (Albany NY). 2018 Jul 19;10(7):1682–97. doi: 10.18632/aging.101501 (PMC6075443; doi:10.18632/aging.101501)
Supplement: Supplementary Table S1 [file aging-10-101501-s001.docx]

**Supplementary Table S1. Proteins which concentration increased at least two times during aging are in blue.**

**Part A. Hippocampus (pages 1-10)**

| **Protein names** | **Gene names** | **t-test Significant** | **-log(10)**  **of p-value** | **Ratio adult/young** | **Razor + unique peptides** | **Young** | | **Adult** | |
| --- | --- | --- | --- | --- | --- | --- | --- | --- | --- |
|  |  |  |  |  |  | **Mean concentration** | **SD** | **Mean concentration** | **SD** |
| Keratin, type II cytoskeletal 8 | Krt8 | + | 4.604025 | **0.073957** | 16 | 0.83246 | 0.334562 | 0.061566 | 0.05273 |
| Unconventional myosin-Vb | Myo5b | + | 1.471628 | **0.089776** | 9 | 0.023429 | 0.018813 | 0.002103 | 0.003893 |
|  | Pcdh17 | + | 5.966088 | **0.098986** | 11 | 0.526599 | 0.059471 | 0.052126 | 0.022121 |
| Fucose mutarotase | Fuom | + | 5.425384 | **0.10107** | 4 | 0.774409 | 0.08673 | 0.07827 | 0.092292 |
| Anion exchange protein 2 | Slc4a2 | + | 2.342038 | **0.101619** | 18 | 0.154359 | 0.121685 | 0.015686 | 0.008046 |
| Coxsackievirus and adenovirus receptor homolog | Cxadr | + | 7.378014 | **0.103568** | 13 | 1.073187 | 0.106532 | 0.111147 | 0.065749 |
| Long-chain-fatty-acid--CoA ligase 4 | Acsl4 | + | 2.263447 | **0.105517** | 19 | 2.77917 | 1.84548 | 0.293251 | 0.088603 |
| Prominin-1 | Prom1 | + | 5.158069 | **0.135733** | 14 | 0.172412 | 0.024708 | 0.023402 | 0.020941 |
| Serpin H1 | Serpinh1 | + | 4.850839 | **0.143906** | 10 | 0.638834 | 0.09242 | 0.091932 | 0.046395 |
| Tenascin | Tnc | + | 11.92109 | **0.148491** | 57 | 2.220333 | 0.169792 | 0.3297 | 0.023211 |
| Uncharacterized family 31 glucosidase KIAA1161 | Kiaa1161 | + | 2.385063 | **0.148983** | 10 | 0.376359 | 0.289041 | 0.056071 | 0.015458 |
| Matrilin-4 | Matn4 | + | 8.652256 | **0.150251** | 16 | 0.736145 | 0.084762 | 0.110607 | 0.021755 |
| Fibulin-5 | Fbln5 | + | 1.608437 | **0.15407** | 8 | 0.255956 | 0.172032 | 0.039435 | 0.042549 |
| Kinase suppressor of Ras 2 | Ksr2 | + | 2.017469 | **0.155918** | 17 | 0.223856 | 0.1848 | 0.034903 | 0.011343 |
| Neuropilin and tolloid-like protein 1 | Neto1 | + | 2.258012 | **0.16425** | 7 | 0.081973 | 0.018501 | 0.013464 | 0.017304 |
| A-kinase anchor protein 1, mitochondrial | Akap1 | + | 2.761248 | **0.189214** | 17 | 0.087967 | 0.042218 | 0.016645 | 0.018297 |
| Caspase-1;Caspase-1 subunit p20;Caspase-1 subunit p10 | Casp1 | + | 3.85725 | **0.19852** | 6 | 0.125286 | 0.049337 | 0.024872 | 0.012633 |
| 2-hydroxyacylsphingosine 1-beta-galactosyltransferase | Ugt8 | + | 5.337853 | **0.20417** | 17 | 0.538811 | 0.118205 | 0.110009 | 0.034587 |
| MARCKS-related protein | Marcksl1 | + | 12.10284 | **0.207468** | 7 | 18.35082 | 0.976625 | 3.8072 | 0.216558 |
| Beta-galactosidase | Glb1 | + | 4.555055 | **0.208966** | 11 | 0.158891 | 0.050361 | 0.033203 | 0.018675 |
| Fatty acid-binding protein, brain | Fabp7 | + | 6.781376 | **0.211887** | 12 | 16.29681 | 1.566464 | 3.453076 | 0.857899 |
| Gamma-butyrobetaine dioxygenase | Bbox1 | + | 1.579031 | **0.212561** | 7 | 0.302532 | 0.266105 | 0.064306 | 0.014598 |
| Leucine-rich repeat-containing protein 16A | Lrrc16a | + | 3.23555 | **0.220202** | 22 | 0.085702 | 0.040649 | 0.018872 | 0.008004 |
| Neuronal migration protein doublecortin | Dcx | + | 2.303027 | **0.22157** | 7 | 0.730808 | 0.357614 | 0.161925 | 0.104877 |
| Dihydropyrimidinase-related protein 3 | Dpysl3 | + | 10.53561 | **0.222078** | 36 | 32.38258 | 2.627516 | 7.191459 | 0.370831 |
| Fatty acid desaturase 2 | Fads2 | + | 5.6905 | **0.229656** | 6 | 0.312262 | 0.048879 | 0.071713 | 0.019989 |
| Fatty acid desaturase 1 | Fads1 | + | 3.586807 | **0.23292** | 8 | 0.341874 | 0.129093 | 0.079629 | 0.029458 |
| Histone H1.5 | Hist1h1b | + | 8.485389 | **0.237586** | 10 | 10.088 | 0.504996 | 2.396764 | 0.35121 |
| Chondroadherin-like protein | Chadl | + | 1.966456 | **0.248672** | 10 | 0.058068 | 0.025209 | 0.01444 | 0.014703 |
| Endonuclease/exonuclease/phosphatase family domain-containing protein 1 | Eepd1 | + | 1.588803 | **0.25405** | 7 | 0.045512 | 0.008283 | 0.011562 | 0.01341 |
| Programmed cell death protein 4 | Pdcd4 | + | 1.759517 | **0.254346** | 11 | 0.405268 | 0.293035 | 0.103078 | 0.048044 |
| Lipid phosphate phosphatase-related protein type 3 | Lppr3 | + | 4.909749 | **0.258378** | 15 | 0.431336 | 0.081084 | 0.111448 | 0.02875 |
| Receptor-type tyrosine-protein phosphatase T;Protein-tyrosine-phosphatase | Ptprt | + | 3.363803 | **0.265537** | 15 | 0.094853 | 0.036562 | 0.025187 | 0.009689 |
| Phosphatidylinositol 3,4,5-trisphosphate 5-phosphatase 2 | Inppl1 | + | 2.089153 | **0.266831** | 20 | 0.074944 | 0.027825 | 0.019997 | 0.017713 |
| Trophoblast glycoprotein | Tpbg | + | 4.155796 | **0.279628** | 5 | 0.224815 | 0.079377 | 0.062864 | 0.008355 |
|  | Uvrag | + | 3.780336 | **0.282681** | 13 | 0.120003 | 0.029134 | 0.033923 | 0.015389 |
| Histone H2B type 1-F/J/L;  Histone H2B type 1-P;  Histone H2B;  Histone H2B type 1-A | Hist1h2bf;  Hist1h2bp;  Hist1h2br;  Hist1h2ba | + | 1.518342 | **0.282866** | 3 | 0.231187 | 0.054723 | 0.065395 | 0.072561 |
| N-acetylgalactosamine kinase | Galk2 | + | 3.701492 | **0.283006** | 11 | 0.913446 | 0.318435 | 0.258511 | 0.051604 |
| Methylthioribulose-1-phosphate dehydratase | Apip | + | 2.136997 | **0.288677** | 3 | 0.241219 | 0.133535 | 0.069634 | 0.060616 |
| Mitogen-activated protein kinase kinase kinase 10 | Map3k10 | + | 2.574417 | **0.295889** | 8 | 0.056167 | 0.008996 | 0.016619 | 0.019771 |
| Calcium-binding protein 39-like | Cab39l | + | 2.429149 | **0.298494** | 12 | 0.635327 | 0.252733 | 0.189641 | 0.102577 |
| Protein transport protein Sec24A | Sec24a | + | 3.337232 | **0.299025** | 14 | 0.109214 | 0.063904 | 0.032658 | 0.005761 |
| Very long-chain specific acyl-CoA dehydrogenase, mitochondrial | Acadvl | + | 8.382519 | **0.305667** | 32 | 8.268375 | 1.015934 | 2.527367 | 0.188094 |
| Ras-related C3 botulinum toxin substrate 3 | Rac3 | + | 5.583047 | **0.305837** | 3 | 1.308152 | 0.296819 | 0.400082 | 0.041501 |
| Dihydropyrimidinase-related protein 5 | Dpysl5 | + | 9.838053 | **0.307038** | 39 | 25.72154 | 1.543047 | 7.897488 | 0.456416 |
| Testis-expressed sequence 10 protein | Tex10 | + | 2.089709 | **0.307984** | 9 | 0.079209 | 0.042477 | 0.024395 | 0.022518 |
| Lysophosphatidylcholine acyltransferase 2 | Lpcat2 | + | 1.603388 | **0.307988** | 6 | 0.151108 | 0.097271 | 0.046539 | 0.037875 |
| Ras-related protein R-Ras2 | Rras2 | + | 10.83831 | **0.308657** | 12 | 6.800617 | 0.45813 | 2.099055 | 0.082137 |
| Pre-mRNA-splicing factor SYF1 | Xab2 | + | 1.835882 | **0.308789** | 8 | 0.080031 | 0.056087 | 0.024713 | 0.012936 |
| AarF domain-containing protein kinase 4 | Adck4 | + | 1.923338 | **0.309872** | 15 | 0.215812 | 0.089422 | 0.066874 | 0.034752 |
| Equilibrative nucleoside transporter 2 | Slc29a2 | + | 2.10018 | **0.310304** | 4 | 0.158258 | 0.035754 | 0.049108 | 0.049473 |
| PHD finger protein 14 | Phf14 | + | 2.90456 | **0.316334** | 7 | 0.068209 | 0.01828 | 0.021577 | 0.011133 |
| cTAGE family member 5 | Ctage5 | + | 1.727969 | **0.317141** | 13 | 0.155826 | 0.108668 | 0.049419 | 0.034517 |
| Repulsive guidance molecule A | Rgma | + | 2.426533 | **0.318533** | 7 | 0.232961 | 0.076067 | 0.074206 | 0.044495 |
| Transthyretin | Ttr | + | 3.38361 | **0.319334** | 5 | 15.54656 | 5.436997 | 4.964541 | 0.664805 |
| Plexin-D1 | Plxnd1 | + | 2.387473 | **0.321452** | 27 | 0.18079 | 0.086646 | 0.058115 | 0.026148 |
| FK506-binding protein-like | Fkbpl | + | 2.146919 | **0.322268** | 5 | 0.094171 | 0.023883 | 0.030348 | 0.026696 |
| Zinc transporter ZIP12 | Slc39a12 | + | 5.620402 | **0.323199** | 9 | 0.334288 | 0.064448 | 0.108041 | 0.017473 |
| Diphosphomevalonate decarboxylase | Mvd | + | 2.942777 | **0.323288** | 9 | 0.149794 | 0.046839 | 0.048427 | 0.029393 |
| Inward rectifier potassium channel 13 | Kcnj13 | + | 1.938401 | **0.324251** | 5 | 0.526374 | 0.223148 | 0.170677 | 0.088243 |
| Multivesicular body subunit 12B | Mvb12b | + | 2.127516 | **0.329852** | 10 | 0.232228 | 0.118523 | 0.076601 | 0.018729 |
| Thymocyte nuclear protein 1 | Thyn1 | + | 1.619841 | **0.330152** | 7 | 0.313042 | 0.125572 | 0.103351 | 0.099612 |
| Regulator of G-protein signaling 8 | Rgs8 | + | 1.531972 | **0.331703** | 7 | 0.275162 | 0.061423 | 0.091272 | 0.121364 |
| ADP-ribosyl cyclase/cyclic ADP-ribose hydrolase 1 | Cd38 | + | 5.820417 | **0.331845** | 8 | 0.773928 | 0.107843 | 0.256824 | 0.049535 |
| Parathymosin | Ptms | + | 1.51328 | **0.335037** | 6 | 8.891978 | 3.548642 | 2.979138 | 3.379652 |
| Nuclear pore complex protein Nup160 | Nup160 | + | 4.978407 | **0.335231** | 19 | 0.342287 | 0.085443 | 0.114745 | 0.014003 |
| 40S ribosomal protein S25 | Rps25 | + | 3.157657 | **0.335786** | 8 | 15.0298 | 6.268734 | 5.046788 | 1.192346 |
| D-beta-hydroxybutyrate dehydrogenase, mitochondrial | Bdh1 | + | 10.67353 | **0.336345** | 21 | 25.73073 | 0.828166 | 8.654399 | 0.495296 |
| Zinc finger and BTB domain-containing protein 18 | Zbtb18 | + | 2.434505 | **0.337693** | 8 | 0.127961 | 0.042181 | 0.043211 | 0.024332 |
| Ubiquitin-associated protein 1 | Ubap1 | + | 1.883404 | **0.338975** | 4 | 0.173303 | 0.044288 | 0.058745 | 0.062194 |
| Cytoplasmic polyadenylation element-binding protein 4 | Cpeb4 | + | 1.992429 | **0.339684** | 8 | 0.056484 | 0.011824 | 0.019187 | 0.021828 |
| Calcium homeostasis endoplasmic reticulum protein | Cherp | + | 3.299031 | **0.342524** | 10 | 0.203983 | 0.061674 | 0.069869 | 0.023638 |
| Mucolipin-1 | Mcoln1 | + | 3.552555 | **0.346886** | 6 | 0.122103 | 0.035896 | 0.042356 | 0.021307 |
| Apolipoprotein A-IV | Apoa4 | + | 2.769388 | **0.347385** | 14 | 0.246678 | 0.085147 | 0.085692 | 0.040722 |
| Mitochondrial inner membrane protein OXA1L | Oxa1l | + | 4.15512 | **0.347894** | 6 | 0.186176 | 0.042521 | 0.064769 | 0.022714 |
| Nuclease-sensitive element-binding protein 1 | Ybx1 | + | 3.958359 | **0.349415** | 12 | 2.319403 | 0.668609 | 0.810433 | 0.144535 |
| Cleavage and polyadenylation specificity factor subunit 2 | Cpsf2 | + | 2.028273 | **0.352128** | 11 | 0.048208 | 0.014399 | 0.016975 | 0.015409 |
| Angiotensin-converting enzyme;  Angiotensin-converting enzyme, soluble form | Ace | + | 2.175029 | **0.353307** | 17 | 0.296574 | 0.148022 | 0.104781 | 0.03937 |
| Interferon-inducible double-stranded RNA-dependent protein kinase activator A | Prkra | + | 1.607602 | **0.354088** | 7 | 0.268499 | 0.13608 | 0.095072 | 0.071378 |
| Kelch-like protein 9 | Klhl9 | + | 2.573476 | **0.354152** | 5 | 0.038865 | 0.031755 | 0.013764 | 0.00827 |
| Bifunctional 3-phosphoadenosine 5-phosphosulfate synthase 1;  Sulfate adenylyltransferase;  Adenylyl-sulfate kinase | Papss1 | + | 4.480401 | **0.35436** | 19 | 0.339806 | 0.092222 | 0.120414 | 0.013048 |
| WD repeat-containing protein 18 | Wdr18 | + | 2.542118 | **0.355495** | 6 | 0.072645 | 0.043383 | 0.025825 | 0.020766 |
| Signal recognition particle receptor subunit alpha | Srpr | + | 2.67999 | **0.355997** | 14 | 0.238009 | 0.081328 | 0.08473 | 0.029411 |
| Torsin-1A | Tor1a | + | 1.560459 | **0.356349** | 7 | 0.144401 | 0.035735 | 0.051457 | 0.063946 |
| Ras-interacting protein 1 | Rasip1 | + | 2.489207 | **0.356364** | 9 | 0.115963 | 0.018113 | 0.041325 | 0.031483 |
| AP-5 complex subunit sigma-1 | Ap5s1 | + | 1.583216 | **0.358549** | 4 | 0.346199 | 0.06594 | 0.124129 | 0.149518 |
| FAD-dependent oxidoreductase domain-containing protein 1 | Foxred1 | + | 4.051233 | **0.360355** | 8 | 0.485526 | 0.136008 | 0.174962 | 0.025697 |
| ELMO domain-containing protein 2 | Elmod2 | + | 2.016775 | **0.361546** | 5 | 0.203206 | 0.051058 | 0.073468 | 0.062712 |
| Serine/threonine-protein phosphatase PGAM5, mitochondrial | Pgam5 | + | 5.400613 | **0.363248** | 19 | 5.73936 | 0.937142 | 2.084809 | 0.357908 |
|  | Calu | + | 2.031128 | **0.363343** | 2 | 0.150144 | 0.030154 | 0.054554 | 0.049094 |
| Mitochondrial Rho GTPase 2 | Rhot2;  Gm20683 | + | 1.514798 | **0.363784** | 12 | 0.15135 | 0.057373 | 0.055059 | 0.048193 |
| Hydroxysteroid dehydrogenase-like protein 2 | Hsdl2 | + | 1.491847 | **0.366285** | 25 | 1.385673 | 0.877382 | 0.507551 | 0.095508 |
| Phosphatidylinositol 3,4,5-trisphosphate-dependent Rac exchanger 1 protein | Prex1 | + | 2.519377 | **0.366375** | 47 | 0.255605 | 0.090374 | 0.093647 | 0.033198 |
| Protein FAM73B | Fam73b | + | 2.680303 | **0.369173** | 9 | 0.241929 | 0.05551 | 0.089314 | 0.052184 |
| 7SK snRNA methylphosphate capping enzyme | Mepce | + | 1.836828 | **0.372298** | 8 | 0.03736 | 0.022932 | 0.013909 | 0.011008 |
| Transmembrane protein 19 | Tmem19 | + | 1.835889 | **0.375875** | 2 | 0.331654 | 0.054498 | 0.12466 | 0.097795 |
| Acyl-CoA dehydrogenase family member 11 | Acad11 | + | 1.914914 | **0.376152** | 14 | 0.104811 | 0.029357 | 0.039425 | 0.027413 |
| Interferon regulatory factor 2-binding protein-like | Irf2bpl | + | 4.688399 | **0.376524** | 18 | 0.063738 | 0.010267 | 0.023999 | 0.005752 |
| Cadherin-8 | Cdh8 | + | 2.676799 | **0.378021** | 9 | 0.112598 | 0.039247 | 0.042564 | 0.009764 |
| Dihydroxyacetone phosphate acyltransferase | Gnpat | + | 2.206286 | **0.379867** | 11 | 0.085384 | 0.027649 | 0.032435 | 0.014604 |
| Protocadherin-19 | Pcdh19 | + | 1.775751 | **0.381469** | 8 | 0.098528 | 0.06434 | 0.037585 | 0.011995 |
| Peroxisome assembly factor 2 | Pex6 | + | 3.12696 | **0.381724** | 9 | 0.055885 | 0.01329 | 0.021333 | 0.010673 |
| Protein scribble homolog | Scrib | + | 3.367655 | **0.383937** | 26 | 0.068115 | 0.01833 | 0.026152 | 0.007512 |
| General transcription factor IIF subunit 2 | Gtf2f2 | + | 1.651221 | **0.385356** | 6 | 0.248033 | 0.080829 | 0.095581 | 0.075053 |
| Beta-catenin-like protein 1 | Ctnnbl1 | + | 3.39161 | **0.387458** | 11 | 0.14413 | 0.029598 | 0.055844 | 0.020265 |
| Microtubule-associated serine/threonine-protein kinase 1 | Mast1 | + | 2.695156 | **0.387587** | 24 | 0.40224 | 0.151918 | 0.155903 | 0.036425 |
| Activity-dependent neuroprotector homeobox protein | Adnp | + | 3.818112 | **0.390409** | 25 | 0.068814 | 0.017443 | 0.026866 | 0.00453 |
| Sialidase-4 | Neu4 | + | 4.468659 | **0.39211** | 4 | 0.134448 | 0.019547 | 0.052718 | 0.012579 |
| Axin interactor, dorsalization-associated protein | Aida | + | 1.488689 | **0.39419** | 11 | 0.769545 | 0.512465 | 0.303347 | 0.051611 |
| Leucine-rich repeat and fibronectin type-III domain-containing protein 4 | Lrfn4 | + | 3.626819 | **0.394607** | 9 | 0.156458 | 0.043926 | 0.06174 | 0.012451 |
| 8-oxo-dGDP phosphatase NUDT18 | Nudt18 | + | 1.981677 | **0.394915** | 7 | 0.187148 | 0.0568 | 0.073908 | 0.057774 |
| Phosphatidylinositol 3,4,5-trisphosphate-dependent Rac exchanger 2 protein | Prex2 | + | 1.745282 | **0.3975** | 23 | 0.02914 | 0.019688 | 0.011583 | 0.008135 |
| Plexin-B3 | Plxnb3 | + | 2.643342 | **0.40025** | 8 | 0.047568 | 0.010271 | 0.019039 | 0.009894 |
| Progressive ankylosis protein | Ankh | + | 3.307312 | **0.400751** | 10 | 0.560749 | 0.215465 | 0.224721 | 0.017458 |
| ELKS/Rab6-interacting/CAST family member 1 | Erc1 | + | 2.061325 | **0.4042** | 32 | 0.60651 | 0.231097 | 0.245151 | 0.168068 |
| Pre-B-cell leukemia transcription factor-interacting protein 1 | Pbxip1 | + | 1.875534 | **0.409146** | 11 | 0.172515 | 0.018375 | 0.070584 | 0.051119 |
| Elongation of very long chain fatty acids protein 5;  Elongation of very long chain fatty acids protein | Elovl5 | + | 1.807738 | **0.409715** | 3 | 0.251016 | 0.10501 | 0.102845 | 0.050802 |
| Pleiotropic regulator 1 | Plrg1 | + | 2.807458 | **0.410823** | 18 | 0.083478 | 0.019153 | 0.034295 | 0.020922 |
| Integrator complex subunit 1 | Ints1 | + | 1.922727 | **0.411229** | 15 | 0.03638 | 0.009236 | 0.014961 | 0.008422 |
| Tyrosine-protein kinase BAZ1B | Baz1b | + | 2.129837 | **0.411952** | 32 | 0.038333 | 0.009395 | 0.015791 | 0.006581 |
| Transcription factor RFX3 | Rfx3 | + | 2.003174 | **0.412541** | 6 | 0.059244 | 0.023094 | 0.024441 | 0.008787 |
| Fatty acyl-CoA reductase 1 | Far1 | + | 3.629428 | **0.416353** | 8 | 0.137848 | 0.020497 | 0.057393 | 0.021414 |
| Semaphorin-4A | Sema4a | + | 3.1222 | **0.41977** | 12 | 0.147242 | 0.030362 | 0.061808 | 0.021808 |
| Dephospho-CoA kinase domain-containing protein | Dcakd | + | 5.464266 | **0.420255** | 9 | 3.706843 | 0.556053 | 1.557821 | 0.205655 |
| SLIT and NTRK-like protein 4 | Slitrk4 | + | 3.367163 | **0.421029** | 6 | 0.0311 | 0.016808 | 0.013094 | 0.003473 |
| N-acetyltransferase 10 | Nat10 | + | 2.442383 | **0.425966** | 11 | 0.045442 | 0.013483 | 0.019357 | 0.006553 |
| 39S ribosomal protein L28, mitochondrial | Mrpl28 | + | 1.996048 | **0.42728** | 7 | 0.264828 | 0.07754 | 0.113156 | 0.03716 |
| BTB/POZ domain-containing protein KCTD3 | Kctd3 | + | 1.845048 | **0.429522** | 6 | 0.029341 | 0.017262 | 0.012603 | 0.011012 |
| Phosphatidylserine synthase 1 | Ptdss1 | + | 2.224471 | **0.43019** | 5 | 0.046513 | 0.011448 | 0.020009 | 0.010531 |
| NADH dehydrogenase [ubiquinone] complex I, assembly factor 7 | Ndufaf7 | + | 2.693397 | **0.435878** | 8 | 0.223491 | 0.063111 | 0.097415 | 0.022508 |
| Histone H1.1 | Hist1h1a | + | 4.329867 | **0.438243** | 6 | 1.66347 | 0.226713 | 0.729004 | 0.146354 |
| Cleavage and polyadenylation specificity factor subunit 1 | Cpsf1 | + | 4.487099 | **0.439284** | 19 | 0.075431 | 0.01136 | 0.033136 | 0.005939 |
| Anaphase-promoting complex subunit 5 | Anapc5 | + | 1.787323 | **0.439882** | 10 | 0.123204 | 0.018007 | 0.054195 | 0.030768 |
| Rho GDP-dissociation inhibitor 2 | Arhgdib | + | 1.807463 | **0.44084** | 6 | 1.744096 | 0.723592 | 0.768868 | 0.15883 |
| Tubulin alpha-1A chain | Tuba1a | + | 2.177862 | **0.442023** | 3 | 1.282855 | 0.629344 | 0.567051 | 0.104207 |
| Mitochondrial ornithine transporter 1 | Slc25a15 | + | 2.249924 | **0.444065** | 7 | 0.116288 | 0.038838 | 0.05164 | 0.026133 |
| Basic leucine zipper and W2 domain-containing protein 2 | Bzw2 | + | 2.881703 | **0.444688** | 14 | 0.607749 | 0.229213 | 0.270258 | 0.041239 |
| Isopentenyl-diphosphate Delta-isomerase 1 | Idi1 | + | 4.052315 | **0.445638** | 12 | 1.906115 | 0.380611 | 0.849438 | 0.111188 |
| tRNA (guanine-N(7)-)-methyltransferase non-catalytic subunit WDR4;  tRNA (guanine-N(7)-)-methyltransferase non-catalytic subunit Wdr4 | Wdr4 | + | 1.563051 | **0.447104** | 5 | 0.068966 | 0.039125 | 0.030835 | 0.018001 |
| Cytoskeleton-associated protein 4 | Ckap4 | + | 5.217768 | **0.447317** | 24 | 1.846793 | 0.214856 | 0.826101 | 0.124429 |
| Adenylate cyclase type 3 | Adcy3 | + | 1.539285 | **0.448601** | 7 | 0.046443 | 0.02098 | 0.020834 | 0.004405 |
| 39S ribosomal protein L19, mitochondrial | Mrpl19 | + | 2.672001 | **0.449192** | 9 | 0.471672 | 0.097879 | 0.211871 | 0.077045 |
| Derlin-1 | Derl1 | + | 2.709948 | **0.449236** | 5 | 0.892681 | 0.280673 | 0.401024 | 0.052403 |
| E3 ubiquitin-protein ligase listerin | Ltn1 | + | 1.889292 | **0.453187** | 13 | 0.085647 | 0.037015 | 0.038814 | 0.008234 |
| Integrin alpha-6;  Integrin alpha-6 heavy chain;  Integrin alpha-6 light chain | Itga6 | + | 3.517012 | **0.457391** | 22 | 0.155271 | 0.040491 | 0.071019 | 0.013909 |
| N(G),N(G)-dimethylarginine dimethylaminohydrolase 2 | Ddah2 | + | 2.355528 | **0.463391** | 16 | 1.290541 | 0.390872 | 0.598024 | 0.180532 |
| Chondroitin sulfate proteoglycan 4 | Cspg4 | + | 4.872005 | **0.463931** | 42 | 0.358887 | 0.053295 | 0.166499 | 0.022889 |
| Carnitine O-palmitoyltransferase 1, liver isoform | Cpt1a | + | 3.745339 | **0.464273** | 17 | 0.785562 | 0.184206 | 0.364715 | 0.035832 |
| Lanosterol synthase | Lss | + | 2.787751 | **0.464583** | 10 | 0.104855 | 0.029818 | 0.048714 | 0.01102 |
| Mitogen-activated protein kinase kinase kinase kinase 4 | Map4k4 | + | 2.820651 | **0.466177** | 23 | 0.202718 | 0.028808 | 0.094502 | 0.029014 |
| Ras GTPase-activating protein-binding protein 1 | G3bp1 | + | 6.236841 | **0.466448** | 12 | 0.424281 | 0.023991 | 0.197905 | 0.020881 |
| Retinol-binding protein 1 | Rbp1 | + | 2.999655 | **0.466788** | 8 | 1.683185 | 0.156852 | 0.78569 | 0.271511 |
| Echinoderm microtubule-associated protein-like 1 | Eml1 | + | 5.442465 | **0.467468** | 23 | 0.437091 | 0.035441 | 0.204326 | 0.029815 |
| Receptor-type tyrosine-protein phosphatase gamma;  Protein-tyrosine-phosphatase | Ptprg | + | 2.738543 | **0.467734** | 13 | 0.160926 | 0.049001 | 0.075271 | 0.014519 |
| Seizure protein 6 | Sez6 | + | 1.866639 | **0.469266** | 7 | 0.115851 | 0.048385 | 0.054365 | 0.020446 |
| Proline-, glutamic acid- and leucine-rich protein 1 | Pelp1 | + | 2.916506 | **0.46973** | 13 | 0.113566 | 0.026599 | 0.053345 | 0.011801 |
| Immunoglobulin superfamily member 21 | Igsf21 | + | 2.39105 | **0.470774** | 10 | 0.753032 | 0.264348 | 0.354508 | 0.056916 |
| BolA-like protein 2 | Bola2 | + | 2.547942 | **0.472687** | 3 | 3.633229 | 0.617864 | 1.717378 | 0.73599 |
| 60S ribosomal protein L18a | Rpl18a | + | 2.308324 | **0.475061** | 11 | 4.0463 | 1.193301 | 1.92224 | 0.141099 |
| BRO1 domain-containing protein BROX | Brox | + | 1.508778 | **0.475564** | 12 | 2.278136 | 0.231128 | 1.083399 | 0.779183 |
| Glutaredoxin-related protein 5, mitochondrial | Glrx5 | + | 3.766027 | **0.478576** | 3 | 2.66178 | 0.569127 | 1.273863 | 0.193584 |
| Fasciculation and elongation protein zeta-1 | Fez1 | + | 2.228515 | **0.479247** | 11 | 0.295846 | 0.082139 | 0.141783 | 0.041863 |
| Multiple epidermal growth factor-like domains protein 8 | Megf8 | + | 1.750281 | **0.4804** | 8 | 0.0084 | 0.004406 | 0.004036 | 0.002627 |
| Transducin beta-like protein 2 | Tbl2 | + | 1.981843 | **0.480557** | 14 | 0.245445 | 0.08538 | 0.11795 | 0.033509 |
| Ras GTPase-activating-like protein IQGAP1 | Iqgap1 | + | 2.511233 | **0.481653** | 43 | 0.186616 | 0.036723 | 0.089884 | 0.027349 |
| Leucine-rich repeat and fibronectin type-III domain-containing protein 3 | Lrfn3 | + | 2.130091 | **0.482285** | 5 | 0.256295 | 0.087447 | 0.123607 | 0.03966 |
| Echinoderm microtubule-associated protein-like 4 | Eml4 | + | 1.724032 | **0.483196** | 22 | 0.473634 | 0.145985 | 0.228858 | 0.097941 |
| U2 snRNP-associated SURP motif-containing protein | U2surp | + | 4.500623 | **0.486221** | 22 | 0.337971 | 0.049895 | 0.164328 | 0.025161 |
| Uncharacterized protein KIAA0930 homolog | 5031439G07Rik | + | 2.034658 | **0.486996** | 9 | 0.186839 | 0.033543 | 0.09099 | 0.039284 |
| Synaptotagmin-13 | Syt13 | + | 1.591203 | **0.488517** | 7 | 0.212467 | 0.066821 | 0.103794 | 0.051907 |
| Roundabout homolog 1 | Robo1 | + | 3.995117 | **0.488574** | 19 | 0.196581 | 0.025724 | 0.096044 | 0.020443 |
| Signal-induced proliferation-associated 1-like protein 2 | Sipa1l2 | + | 2.324978 | **0.489816** | 22 | 0.053913 | 0.015383 | 0.026408 | 0.007393 |
| Low-density lipoprotein receptor-related protein 1B | Lrp1b | + | 1.953682 | **0.489977** | 17 | 0.05384 | 0.018749 | 0.02638 | 0.007601 |
| Selenide, water dikinase 1 | Sephs1 | + | 2.087868 | **0.491027** | 11 | 0.424778 | 0.123195 | 0.208578 | 0.072922 |
| Angiotensinogen;  Angiotensin-1;  Angiotensin-2;  Angiotensin-3;  Angiotensin-4;  Angiotensin 1-9;  Angiotensin 1-7;  Angiotensin 1-5;  Angiotensin 1-4 | Agt | + | 1.591472 | **0.492192** | 4 | 0.086406 | 0.047755 | 0.042528 | 0.007465 |
|  | Sbf2 | + | 1.587074 | **0.492369** | 17 | 0.056741 | 0.010546 | 0.027938 | 0.011511 |
| Tyrosine-protein kinase CSK | Csk | + | 2.504032 | **0.494373** | 13 | 0.178744 | 0.045635 | 0.088366 | 0.023101 |
| Calmegin | Clgn | + | 1.928023 | **0.496894** | 12 | 0.235048 | 0.112604 | 0.116794 | 0.019918 |
| Core histone macro-H2A.2 | H2afy2 | + | 3.504504 | **0.497039** | 17 | 1.664895 | 0.268562 | 0.827517 | 0.155706 |
| CUB and sushi domain-containing protein 1 | Csmd1 | + | 3.052189 | **0.497752** | 17 | 0.035161 | 0.005917 | 0.017502 | 0.004397 |
| RNA polymerase II-associated protein 1 | Rpap1 | + | 1.776989 | **0.498356** | 10 | 0.032279 | 0.005807 | 0.016087 | 0.009626 |
| Gem-associated protein 5 | Gemin5 | + | 1.862568 | **0.498604** | 18 | 0.273135 | 0.091626 | 0.136186 | 0.037696 |
| Autophagy protein 5 | Atg5 | + | 2.002267 | **0.499006** | 9 | 0.276235 | 0.083151 | 0.137843 | 0.032106 |
| Roundabout homolog 2 | Robo2 | + | 3.631766 | **0.499875** | 37 | 0.710388 | 0.14988 | 0.355105 | 0.050434 |
| Sodium/potassium/calcium exchanger 4 | Slc24a4 | + | 2.105788 | **2.003289** | 4 | 0.042693 | 0.026656 | 0.085527 | 0.020752 |
| Cdc42 effector protein 4 | Cdc42ep4 | + | 1.955053 | **2.010832** | 17 | 0.163598 | 0.139881 | 0.328968 | 0.091578 |
| Immunity-related GTPase family M protein 1 | Irgm1 | + | 2.08698 | **2.022014** | 10 | 0.07066 | 0.053328 | 0.142876 | 0.04457 |
| Protein S100-B | S100b | + | 4.645941 | **2.030242** | 4 | 2.888062 | 0.55062 | 5.863465 | 0.954711 |
| Integrin beta-2;Integrin beta | Itgb2 | + | 2.400763 | **2.042417** | 6 | 0.025035 | 0.019984 | 0.051133 | 0.005176 |
| Complement C1q subcomponent subunit C | C1qc | + | 4.68199 | **2.042686** | 6 | 0.951933 | 0.157112 | 1.9445 | 0.450255 |
| Ribonuclease UK114 | Hrsp12 | + | 4.937209 | **2.04438** | 9 | 3.79704 | 0.759041 | 7.762594 | 1.375934 |
| NEDD8 | Nedd8 | + | 3.054376 | **2.052104** | 3 | 2.385766 | 0.306514 | 4.89584 | 1.458324 |
| Guanine nucleotide-binding protein G(I)/G(S)/G(O) subunit gamma-5 | Gng5 | + | 2.549739 | **2.057394** | 5 | 0.676383 | 0.27137 | 1.391586 | 0.343006 |
| Osteoclast-stimulating factor 1 | Ostf1 | + | 2.608069 | **2.067431** | 8 | 0.292438 | 0.086992 | 0.604596 | 0.215956 |
| MICOS complex subunit Mic10 | Minos1 | + | 1.494168 | **2.068223** | 4 | 1.463952 | 1.449 | 3.027779 | 0.808428 |
| Myeloid leukemia factor 2 | Mlf2 | + | 4.619946 | **2.069559** | 6 | 1.434297 | 0.370131 | 2.968362 | 0.268107 |
| Iron-sulfur cluster co-chaperone protein HscB, mitochondrial | Hscb | + | 2.220131 | **2.081542** | 8 | 0.157 | 0.100741 | 0.326803 | 0.113063 |
| Alpha-(1,6)-fucosyltransferase | Fut8 | + | 2.182665 | **2.119058** | 9 | 0.038294 | 0.034268 | 0.081146 | 0.018754 |
| UPF0183 protein C16orf70 homolog | | + | 2.771751 | **2.124067** | **9** | 0.083274 | 0.035087 | 0.176879 | 0.03739 |
| Protein C9orf72 homolog | 3110043O21Rik | + | 1.492991 | **2.13574** | 4 | 0.021816 | 0.024862 | 0.046593 | 0.008931 |
| Ubiquinone biosynthesis monooxygenase COQ6, mitochondrial | Coq6 | + | 4.97824 | **2.136231** | 13 | 0.586589 | 0.147524 | 1.25309 | 0.086407 |
| Mycophenolic acid acyl-glucuronide esterase, mitochondrial | Abhd10 | + | 3.236807 | **2.136752** | 15 | 0.214635 | 0.072331 | 0.458622 | 0.098821 |
| 3-keto-steroid reductase | Hsd17b7 | + | 2.009094 | **2.158553** | 7 | 0.078996 | 0.048502 | 0.170518 | 0.08875 |
| Iron-sulfur protein NUBPL | Nubpl | + | 2.288367 | **2.161296** | 4 | 0.086351 | 0.050377 | 0.18663 | 0.050666 |
|  |  | + | 3.113528 | **2.188398** | 4 | 0.418933 | 0.174253 | 0.916793 | 0.053191 |
| Acylpyruvase FAHD1, mitochondrial | Fahd1 | + | 4.687607 | **2.248922** | 13 | 0.937314 | 0.263898 | 2.107945 | 0.196341 |
| Haloacid dehalogenase-like hydrolase domain-containing protein 3 | Hdhd3 | + | 6.173069 | **2.304908** | 11 | 1.932602 | 0.25536 | 4.45447 | 0.798904 |
| Protein FAM81A | Fam81a | + | 2.377333 | **2.328284** | 11 | 0.151371 | 0.055079 | 0.352436 | 0.206425 |
| L-2-hydroxyglutarate dehydrogenase, mitochondrial | L2hgdh | + | 4.553381 | **2.339437** | 16 | 0.315028 | 0.086898 | 0.736989 | 0.125142 |
| Junctophilin-1 | Jph1 | + | 1.505888 | **2.354382** | 10 | 0.086804 | 0.02262 | 0.20437 | 0.157808 |
| Ubiquinone biosynthesis O-methyltransferase, mitochondrial | Coq3 | + | 4.676269 | **2.367258** | 7 | 0.378223 | 0.089588 | 0.895352 | 0.120434 |
| MICAL-like protein 1 | Micall1 | + | 2.063189 | **2.375141** | 6 | 0.074587 | 0.083802 | 0.177155 | 0.028597 |
| Citrate synthase | Csl | + | 3.034851 | **2.41565** | 3 | 0.041576 | 0.032975 | 0.100433 | 0.008657 |
| 1,2-dihydroxy-3-keto-5-methylthiopentene dioxygenase | Adi1 | + | 1.75009 | **2.439393** | 7 | 0.394334 | 0.221824 | 0.961935 | 0.248473 |
| Acid ceramidase;Acid ceramidase subunit alpha;Acid ceramidase subunit beta | Asah1 | + | 4.60884 | **2.486436** | 14 | 0.458044 | 0.11246 | 1.138896 | 0.260936 |
| Complement C1q subcomponent subunit A | C1qa | + | 2.846557 | **2.487355** | 8 | 0.559211 | 0.271323 | 1.390957 | 0.296812 |
| Plastin-1 | Pls1 | + | 5.817373 | **2.489668** | 23 | 0.148113 | 0.027053 | 0.368753 | 0.053464 |
| Pre-mRNA-processing factor 40 homolog B | Prpf40b | + | 2.151413 | **2.515864** | 9 | 0.097371 | 0.124895 | 0.244973 | 0.036743 |
| CD82 antigen;Tetraspanin | Cd82 | + | 6.979943 | **2.54564** | 5 | 1.160318 | 0.096034 | 2.953752 | 0.480556 |
| EGF-like repeat and discoidin I-like domain-containing protein 3 | Edil3 | + | 1.730809 | **2.553119** | 7 | 0.047496 | 0.053527 | 0.121263 | 0.04267 |
| Aggrecan core protein | Acan | + | 7.685722 | **2.573291** | 17 | 0.104281 | 0.014633 | 0.268344 | 0.013231 |
| E3 ubiquitin-protein ligase ARIH2 | Arih2 | + | 2.665242 | **2.576501** | 3 | 0.141351 | 0.04835 | 0.36419 | 0.163878 |
| Costars family protein ABRACL | Abracl | + | 2.308185 | **2.581829** | 4 | 0.97819 | 0.708204 | 2.52552 | 0.777106 |
| Microtubule-associated protein tau | Mapt | + | 2.002718 | **2.636688** | 2 | 0.117858 | 0.102776 | 0.310754 | 0.059379 |
| Tubulin alpha-8 chain | Tuba8 | + | 4.88736 | **2.670432** | 11 | 0.627731 | 0.188898 | 1.676313 | 0.152467 |
| Stannin | Snn | + | 2.175392 | **2.679761** | 3 | 0.395772 | 0.456033 | 1.060573 | 0.129056 |
| Aromatic-L-amino-acid decarboxylase | Ddc | + | 5.641624 | **2.751387** | 15 | 0.400317 | 0.1081 | 1.101426 | 0.138532 |
| Pleckstrin homology domain-containing family B member 1 | Plekhb1 | + | 2.942201 | **2.772821** | 7 | 0.266501 | 0.211183 | 0.73896 | 0.094413 |
| Rab GTPase-binding effector protein 2 | Rabep2 | + | 2.715921 | **2.786074** | 10 | 0.051807 | 0.049028 | 0.144339 | 0.02004 |
| FYVE, RhoGEF and PH domain-containing protein 4 | Fgd4 | + | 2.171276 | **2.812507** | 11 | 0.067352 | 0.027095 | 0.189429 | 0.084124 |
| Integrator complex subunit 10 | Ints10 | + | 1.929741 | **2.822086** | 6 | 0.032553 | 0.040328 | 0.091867 | 0.028836 |
| Nuclear receptor corepressor 1 | Ncor1 | + | 1.717574 | **2.950548** | 9 | 0.048676 | 0.040088 | 0.143622 | 0.062972 |
| Inter-alpha-trypsin inhibitor heavy chain H3 | Itih3 | + | 5.402017 | **3.025631** | 10 | 0.045817 | 0.006458 | 0.138624 | 0.038084 |
| Urea transporter 1 | Slc14a1 | + | 3.682044 | **3.089288** | 6 | 0.096283 | 0.065039 | 0.297447 | 0.068448 |
| WW domain-containing oxidoreductase | Wwox | + | 1.995454 | **3.089482** | 6 | 1.55385 | 1.956843 | 4.800593 | 0.817577 |
| G-protein coupled receptor-associated sorting protein 2 | Gprasp2 | + | 1.915887 | **3.100842** | 10 | 0.100442 | 0.021923 | 0.311455 | 0.176422 |
| Alpha-crystallin B chain | Cryab | + | 5.123748 | **3.112709** | 10 | 3.071438 | 0.980213 | 9.560493 | 1.855034 |
| Carbonic anhydrase 1 | Ca1 | + | 6.645348 | **3.219325** | 11 | 0.260475 | 0.069515 | 0.838552 | 0.127816 |
| Calcineurin B homologous protein 3 | Tesc | + | 2.578455 | **3.31567** | 7 | 0.110659 | 0.087023 | 0.36691 | 0.142454 |
|  | Mdn1 | + | 2.370854 | **3.329952** | 15 | 0.006349 | 0.005266 | 0.021141 | 0.011129 |
| CD151 antigen | Cd151 | + | 1.574035 | **3.41261** | 4 | 0.054191 | 0.088539 | 0.184934 | 0.020419 |
| Aspartoacylase | Aspa | + | 4.961733 | **3.494198** | 13 | 0.336015 | 0.094042 | 1.174101 | 0.332765 |
| Palmitoyl-protein thioesterase 1 | Ppt1 | + | 5.220708 | **3.53146** | 8 | 0.502333 | 0.167964 | 1.77397 | 0.268462 |
| Spermatogenesis-associated protein 13 | Spata13 | + | 1.644852 | **3.583338** | 2 | 0.018189 | 0.020669 | 0.065177 | 0.038751 |
| Aldehyde dehydrogenase family 3 member B1 | Aldh3b1 | + | 6.136008 | **3.658955** | 14 | 0.394642 | 0.087506 | 1.443977 | 0.295734 |
| Myelin-associated oligodendrocyte basic protein | Mobp | + | 7.237965 | **3.75581** | 19 | 5.792427 | 1.196235 | 21.75525 | 2.43628 |
| Serine protease HTRA1 | Htra1 | + | 5.824496 | **3.94937** | 11 | 0.073426 | 0.01205 | 0.289987 | 0.084822 |
| Tubulointerstitial nephritis antigen-like | Tinagl1 | + | 4.029208 | **4.127501** | 8 | 0.122621 | 0.035812 | 0.506118 | 0.212579 |
| Isovaleryl-CoA dehydrogenase, mitochondrial | Ivd | + | 10.38453 | **4.302047** | 22 | 1.092952 | 0.117493 | 4.701932 | 0.322159 |
| Leukocyte elastase inhibitor A | Serpinb1a | + | 7.449487 | **4.419483** | 19 | 0.620259 | 0.149772 | 2.741225 | 0.252858 |
| H-2 class I histocompatibility antigen, K-D alpha chain | H2-K1 | + | 1.953929 | **4.999875** | 9 | 0.1336 | 0.058054 | 0.667986 | 0.314289 |
| Actin-binding protein anillin | Anln | + | 5.90636 | **5.468946** | 22 | 0.063785 | 0.024385 | 0.348836 | 0.050317 |
| Phosphoinositide phospholipase C | Plcb4 | + | 3.395824 | **7.380637** | 46 | 0.026861 | 0.010409 | 0.198251 | 0.090235 |
| Ig gamma-2B chain C region | Ighg2b;Igh-3 | + | 4.259946 | **7.492381** | 12 | 0.231972 | 0.144941 | 1.738022 | 0.246971 |
| Cytochrome c oxidase subunit 7A-related protein, mitochondrial | Cox7a2l | + | 3.729098 | **8.156607** | 6 | 1.459163 | 0.310284 | 11.90182 | 5.943506 |
| Hyaluronan and proteoglycan link protein 2 | Hapln2 | + | 8.676086 | **21.14847** | 15 | 0.12854 | 0.040647 | 2.718423 | 0.414332 |

**Part B. Cerebellum (pages 11-19)**

| **Protein**  **names** | **Gene**  **names** | **t-test Significant** | **-log(10) of p-value** | **Ratio adult/young** | **Razor + unique peptides** | **Young** | | **Adult** | |
| --- | --- | --- | --- | --- | --- | --- | --- | --- | --- |
|  |  |  |  |  |  | **Mean concentration** | **SD** | **Mean concentration** | **SD** |
| A-kinase anchor protein 5 | Akap5 | + | 2.782434 | **0.078343** | 28 | 0.627023 | 0.602601 | 0.049123 | 0.030043 |
| Fatty acid-binding protein, brain | Fabp7 | + | 8.817867 | **0.080725** | 12 | 79.62841 | 9.852709 | 6.427987 | 1.738196 |
| DNA (cytosine-5)-methyltransferase 3A | Dnmt3a | + | 7.93087 | **0.100595** | 18 | 0.624907 | 0.134141 | 0.062863 | 0.016161 |
| E3 SUMO-protein ligase CBX4 | Cbx4 | + | 2.677994 | **0.102837** | 4 | 0.086996 | 0.033143 | 0.008946 | 0.010265 |
| High mobility group protein B3 | Hmgb3 | + | 3.933905 | **0.107876** | 3 | 4.537095 | 1.369714 | 0.489443 | 0.348112 |
| General transcription factor 3C polypeptide 5 | Gtf3c5 | + | 4.014614 | **0.119573** | 8 | 0.632049 | 0.169335 | 0.075576 | 0.101174 |
| Prominin-1 | Prom1 | + | 4.356249 | **0.126303** | 14 | 0.14532 | 0.022869 | 0.018354 | 0.009906 |
| Plexin-D1 | Plxnd1 | + | 1.93155 | **0.127999** | 27 | 0.130233 | 0.086471 | 0.01667 | 0.007472 |
| High mobility group protein B2 | Hmgb2 | + | 5.053718 | **0.140696** | 6 | 11.42743 | 2.392486 | 1.607798 | 0.715021 |
| Mediator of RNA polymerase II transcription subunit 24 | Med24 | + | 1.915164 | **0.152659** | 5 | 0.06526 | 0.027188 | 0.009963 | 0.017222 |
| 2-hydroxyacylsphingosine 1-beta-galactosyltransferase | Ugt8 | + | 2.877603 | **0.162962** | 17 | 1.234639 | 0.271253 | 0.2012 | 0.177081 |
| Brain acid soluble protein 1 | Basp1 | + | 4.208525 | **0.163402** | 33 | 57.18957 | 17.17079 | 9.344869 | 6.045163 |
|  | Pcdh17 | + | 2.288953 | **0.166784** | 11 | 0.111132 | 0.13361 | 0.018535 | 0.005764 |
| Mediator of RNA polymerase II transcription subunit 1 | Med1 | + | 2.162776 | **0.169804** | 6 | 0.027261 | 0.007512 | 0.004629 | 0.005352 |
| Graves’ disease carrier protein homolog | Slc25a16 | + | 1.940513 | **0.194952** | 8 | 0.107753 | 0.078831 | 0.021007 | 0.018375 |
| Histone lysine demethylase PHF8 | Phf8 | + | 2.617226 | **0.209085** | 5 | 0.205893 | 0.0755 | 0.043049 | 0.049256 |
| Dihydropyrimidinase-related protein 3 | Dpysl3 | + | 7.231565 | **0.217203** | 36 | 9.882013 | 2.026245 | 2.1464 | 0.292291 |
| NADH dehydrogenase [ubiquinone] complex I, assembly factor 7 | Ndufaf7 | + | 2.59279 | **0.21838** | 8 | 0.287229 | 0.15349 | 0.062725 | 0.05013 |
| Plasminogen receptor (KT) | Plgrkt | + | 3.473154 | **0.221115** | 4 | 1.23639 | 0.179246 | 0.273385 | 0.247807 |
| Dynein light chain Tctex-type 1 | Dynlt1 | + | 4.467313 | **0.222287** | 4 | 0.766354 | 0.210194 | 0.170351 | 0.102643 |
| Tyrosine-protein phosphatase non-receptor type 12 | Ptpn12 | + | 2.190437 | **0.223086** | 7 | 0.048511 | 0.026281 | 0.010822 | 0.008957 |
| Lanosterol 14-alpha demethylase | Cyp51a1 | + | 2.21305 | **0.227381** | 14 | 1.508429 | 1.192546 | 0.342988 | 0.097431 |
| Bridging integrator 2 | Bin2 | + | 2.212582 | **0.231813** | 6 | 0.079046 | 0.030411 | 0.018324 | 0.020495 |
| Coxsackievirus and adenovirus receptor homolog | Cxadr | + | 3.492152 | **0.231996** | 13 | 0.27325 | 0.108761 | 0.063393 | 0.044768 |
| Putative lipoyltransferase 2, mitochondrial | Lipt2 | + | 1.915484 | **0.248915** | 4 | 0.719414 | 0.34156 | 0.179073 | 0.228214 |
| Activating transcription factor 7-interacting protein 1 | Atf7ip | + | 4.706288 | **0.249565** | 9 | 0.044886 | 0.007706 | 0.011202 | 0.004753 |
| Abhydrolase domain-containing protein 1 | Abhd1 | + | 3.598633 | **0.251253** | 3 | 0.202928 | 0.052328 | 0.050986 | 0.04399 |
| Fatty acyl-CoA reductase 1 | Far1 | + | 3.383669 | **0.252585** | 8 | 0.229655 | 0.087174 | 0.058007 | 0.038605 |
| Protein SMG9 | Smg9 | + | 2.885004 | **0.254399** | 4 | 0.151212 | 0.038445 | 0.038468 | 0.043457 |
| Stress-associated endoplasmic reticulum protein 2 | Serp2 | + | 2.068341 | **0.264185** | 3 | 1.778046 | 0.91114 | 0.469734 | 0.392471 |
| Lysine-specific demethylase 3B | Kdm3b | + | 1.972861 | **0.265067** | 8 | 0.079937 | 0.078213 | 0.021189 | 0.008994 |
| Mitochondrial 10-formyltetrahydrofolate dehydrogenase | Aldh1l2 | + | 4.017188 | **0.267205** | 17 | 0.264898 | 0.086009 | 0.070782 | 0.030583 |
| Serine/threonine-protein phosphatase 2A 65 kDa regulatory subunit A beta isoform | Ppp2r1b | + | 3.580647 | **0.276955** | 4 | 0.093537 | 0.009616 | 0.025905 | 0.021531 |
| mRNA-decapping enzyme 1A | Dcp1a | + | 2.206081 | **0.277899** | 6 | 0.090702 | 0.035957 | 0.025206 | 0.022548 |
| Immunoglobulin superfamily member 21 | Igsf21 | + | 3.072895 | **0.281574** | 10 | 1.539624 | 0.523399 | 0.433518 | 0.199384 |
| Proline-rich AKT1 substrate 1 | Akt1s1 | + | 2.223315 | **0.287563** | 5 | 0.315622 | 0.098033 | 0.090761 | 0.096577 |
| DNA ligase 3;DNA ligase | Lig3 | + | 2.951097 | **0.290709** | 13 | 0.088942 | 0.04074 | 0.025856 | 0.010706 |
| Trimeric intracellular cation channel type A | Tmem38a | + | 2.065512 | **0.293393** | 7 | 0.82272 | 0.298936 | 0.24138 | 0.214708 |
| General transcription factor IIH subunit 2 | Gtf2h2 | + | 1.946927 | **0.30253** | 4 | 0.101247 | 0.069402 | 0.03063 | 0.021554 |
| Protein-tyrosine kinase 2-beta | Ptk2b | + | 2.159039 | **0.304073** | 55 | 0.205138 | 0.174317 | 0.062377 | 0.020384 |
| MARCKS-related protein | Marcksl1 | + | 4.527987 | **0.304464** | 7 | 12.2685 | 3.883576 | 3.735316 | 0.739975 |
| Hydroxymethylglutaryl-CoA synthase, cytoplasmic | Hmgcs1 | + | 4.769796 | **0.307325** | 15 | 1.935069 | 0.40472 | 0.594694 | 0.167552 |
| Tenascin | Tnc | + | 7.020451 | **0.313079** | 57 | 2.113154 | 0.17938 | 0.661584 | 0.108123 |
| Y-box-binding protein 3 | Ybx3 | + | 3.263052 | **0.314344** | 8 | 0.975118 | 0.345654 | 0.306523 | 0.07602 |
| Nectin-1 | Pvrl1 | + | 2.1825 | **0.319641** | 8 | 0.655981 | 0.103192 | 0.209678 | 0.16327 |
| Protein phosphatase 1B | Ppm1b | + | 2.086823 | **0.321446** | 9 | 0.489766 | 0.260289 | 0.157433 | 0.068745 |
| PHD finger protein 20-like protein 1 | Phf20l1 | + | 2.684739 | **0.328353** | 4 | 0.122556 | 0.038047 | 0.040241 | 0.025252 |
| Pleckstrin homology domain-containing family A member 1 | Plekha1 | + | 3.016249 | **0.33195** | 10 | 0.362951 | 0.146242 | 0.120481 | 0.062192 |
| Chromobox protein homolog 8 | Cbx8 | + | 2.130972 | **0.333354** | 4 | 0.127021 | 0.069819 | 0.042343 | 0.029159 |
| Sterol-4-alpha-carboxylate 3-dehydrogenase, decarboxylating | Nsdhl | + | 6.86115 | **0.333389** | 12 | 1.082622 | 0.144131 | 0.360934 | 0.044544 |
| 7-dehydrocholesterol reductase | Dhcr7 | + | 4.232452 | **0.333498** | 4 | 1.006347 | 0.265602 | 0.335614 | 0.093743 |
| Translocation protein SEC62 | Sec62 | + | 2.065324 | **0.334894** | 6 | 0.517202 | 0.255219 | 0.173208 | 0.077843 |
| Telomere-associated protein RIF1 | Rif1 | + | 3.25529 | **0.336404** | 16 | 0.065058 | 0.019901 | 0.021886 | 0.007918 |
| Histone H1.1 | Hist1h1a | + | 5.199359 | **0.336527** | 6 | 25.38421 | 4.73116 | 8.542477 | 1.644415 |
| CTTNBP2 N-terminal-like protein | Cttnbp2nl | + | 2.132255 | **0.337767** | 6 | 0.091149 | 0.049704 | 0.030787 | 0.018671 |
| Diphosphomevalonate decarboxylase | Mvd | + | 3.665009 | **0.33819** | 9 | 0.433912 | 0.079243 | 0.146745 | 0.046467 |
| Laminin subunit beta-1 | Lamb1 | + | 2.764842 | **0.349704** | 18 | 0.103118 | 0.031046 | 0.036061 | 0.020603 |
| TBC1 domain family member 23 | Tbc1d23 | + | 2.013126 | **0.353518** | 9 | 0.185224 | 0.073909 | 0.06548 | 0.063357 |
| Ras-related protein R-Ras2 | Rras2 | + | 3.292997 | **0.357074** | 12 | 2.43321 | 0.801512 | 0.868836 | 0.224038 |
| Kin of IRRE-like protein 3;Processed kin of IRRE-like protein 3 | Kirrel3 | + | 3.575605 | **0.358779** | 8 | 0.170963 | 0.045681 | 0.061338 | 0.015584 |
| Prostaglandin F2 receptor negative regulator | Ptgfrn | + | 2.296721 | **0.360018** | 14 | 0.108784 | 0.016898 | 0.039164 | 0.032822 |
| Beta-enolase;Enolase | Eno3 | + | 3.417149 | **0.362211** | 7 | 0.166425 | 0.046012 | 0.060281 | 0.021558 |
| Isoamyl acetate-hydrolyzing esterase 1 homolog | Iah1 | + | 2.356394 | **0.372148** | 5 | 0.355106 | 0.121693 | 0.132152 | 0.074025 |
| Epidermal growth factor receptor kinase substrate 8 | Eps8 | + | 1.974013 | **0.37263** | 18 | 0.389415 | 0.193507 | 0.145108 | 0.086506 |
|  | Jakmip2 | + | 1.866643 | **0.375475** | 13 | 0.079143 | 0.036495 | 0.029716 | 0.023466 |
| Poly(A) polymerase alpha | Papola | + | 2.101566 | **0.379597** | 7 | 0.081824 | 0.032184 | 0.03106 | 0.019894 |
| Ankyrin repeat domain-containing protein 17 | Ankrd17 | + | 3.694929 | **0.381378** | 20 | 0.084253 | 0.017637 | 0.032132 | 0.008925 |
| Chromobox protein homolog 6 | Cbx6 | + | 2.217979 | **0.382153** | 6 | 0.506771 | 0.236049 | 0.193664 | 0.044178 |
| AN1-type zinc finger protein 5 | Zfand5 | + | 2.106914 | **0.387374** | 4 | 0.210808 | 0.060061 | 0.081661 | 0.063772 |
| E3 ubiquitin-protein ligase SHPRH | Shprh | + | 2.350668 | **0.388242** | 15 | 0.067624 | 0.023808 | 0.026254 | 0.016386 |
| Nuclease-sensitive element-binding protein 1 | Ybx1 | + | 3.844008 | **0.390517** | 12 | 3.098291 | 0.850588 | 1.209934 | 0.17558 |
| Histone H1.2 | Hist1h1c | + | 3.069499 | **0.391397** | 6 | 17.99988 | 6.2054 | 7.045107 | 2.297947 |
|  | Uvrag | + | 2.565599 | **0.391441** | 13 | 0.21498 | 0.078052 | 0.084152 | 0.032659 |
| Arf-GAP with coiled-coil, ANK repeat and PH domain-containing protein 2 | Acap2 | + | 2.799136 | **0.397663** | 21 | 0.225156 | 0.060181 | 0.089536 | 0.044884 |
| 60S ribosomal protein L11 | Rpl11;  Gm10036 | + | 4.208317 | **0.399041** | 13 | 13.16927 | 2.981732 | 5.255078 | 1.085687 |
| Protein FAM177A1 | Fam177a1 | + | 3.96498 | **0.399128** | 5 | 1.63191 | 0.130351 | 0.651341 | 0.201976 |
| Heparan sulfate 2-O-sulfotransferase 1 | Hs2st1 | + | 1.901265 | **0.40496** | 4 | 0.379635 | 0.08386 | 0.153737 | 0.142141 |
| Beta-galactosidase | Glb1 | + | 2.786384 | **0.405486** | 11 | 0.231896 | 0.077039 | 0.094031 | 0.017938 |
| Serpin H1 | Serpinh1 | + | 4.419472 | **0.40828** | 10 | 1.008658 | 0.198592 | 0.411815 | 0.069047 |
| Bifunctional 3-phosphoadenosine 5-phosphosulfate synthase 1;  Sulfate adenylyltransferase;  Adenylyl-sulfate kinase | Papss1 | + | 3.779382 | **0.410285** | 19 | 0.938073 | 0.177467 | 0.384877 | 0.085888 |
| Unconventional myosin-Ib | Myo1b | + | 2.497387 | **0.410634** | 30 | 0.381588 | 0.118922 | 0.156693 | 0.08106 |
| Nuclear valosin-containing protein-like | Nvl | + | 2.029279 | **0.410764** | 7 | 0.054061 | 0.019704 | 0.022206 | 0.012187 |
| LysM and putative peptidoglycan-binding domain-containing protein 1 | Lysmd1 | + | 2.140824 | **0.410944** | 8 | 0.733993 | 0.259211 | 0.30163 | 0.13255 |
| 39S ribosomal protein L17, mitochondrial | Mrpl17 | + | 1.871044 | **0.414724** | 6 | 0.38728 | 0.139172 | 0.160614 | 0.146733 |
| Etoposide-induced protein 2.4 | Ei24 | + | 2.124904 | **0.415579** | 10 | 0.326012 | 0.075065 | 0.135484 | 0.076913 |
| Pro-interleukin-16;  Interleukin-16 | Il16 | + | 2.396336 | **0.417332** | 13 | 0.153546 | 0.052113 | 0.06408 | 0.022432 |
| Glycosylphosphatidylinositol anchor attachment 1 protein | Gpaa1 | + | 3.467063 | **0.420263** | 11 | 0.365559 | 0.095089 | 0.153631 | 0.033819 |
| WD repeat domain phosphoinositide-interacting protein 1 | Wipi1 | + | 3.716575 | **0.420382** | 5 | 0.327339 | 0.076036 | 0.137608 | 0.030765 |
| XK-related protein 6;  XK-related protein | Xkr6 | + | 2.065475 | **0.421946** | 5 | 0.065695 | 0.015599 | 0.02772 | 0.017457 |
|  | Arid1b | + | 1.99863 | **0.422119** | 20 | 0.097716 | 0.041792 | 0.041248 | 0.016007 |
| Plakophilin-3 | Pkp3 | + | 2.373686 | **0.424645** | 12 | 0.149011 | 0.048625 | 0.063277 | 0.020442 |
| Copine-9 | Cpne9 | + | 1.941102 | **0.425761** | 10 | 1.136327 | 0.224733 | 0.483803 | 0.221866 |
| G protein-regulated inducer of neurite outgrowth 3 | Gprin3 | + | 2.725652 | **0.430375** | 16 | 0.359127 | 0.100392 | 0.154559 | 0.051618 |
| 60S ribosomal protein L29 | Rpl29;  Gm17669;  Gm10709;  Gm3550 | + | 1.8569 | **0.430542** | 11 | 3.754662 | 2.261484 | 1.61654 | 0.436544 |
| AarF domain-containing protein kinase 4 | Adck4 | + | 2.081673 | **0.430909** | 15 | 0.373498 | 0.062928 | 0.160944 | 0.093995 |
| Putative E3 ubiquitin-protein ligase UBR7 | Ubr7 | + | 1.961804 | **0.436781** | 4 | 0.119402 | 0.04343 | 0.052153 | 0.026561 |
| Cytochrome P450 20A1 | Cyp20a1 | + | 2.959319 | **0.438817** | 7 | 0.211183 | 0.071336 | 0.092671 | 0.023282 |
| cAMP-specific 3,5-cyclic phosphodiesterase 4D | Pde4d | + | 3.73691 | **0.441459** | 15 | 0.475597 | 0.104739 | 0.209956 | 0.041422 |
| Isocitrate dehydrogenase [NADP] cytoplasmic | Idh1 | + | 2.198801 | **0.443838** | 27 | 6.317679 | 1.728802 | 2.804026 | 1.082091 |
| Neuron-specific protein family member 1 | Nsg1 | + | 1.975937 | **0.446951** | 5 | 1.220196 | 0.312486 | 0.545368 | 0.421517 |
| Zinc finger and BTB domain-containing protein 18 | Zbtb18 | + | 4.208774 | **0.447619** | 8 | 0.479757 | 0.084389 | 0.214748 | 0.038645 |
| 39S ribosomal protein L47, mitochondrial | Mrpl47 | + | 1.976255 | **0.45025** | 9 | 0.733597 | 0.195889 | 0.330302 | 0.219761 |
| Phosphatidylinositol transfer protein beta isoform | Pitpnb | + | 2.561313 | **0.4503** | 12 | 1.737902 | 0.598296 | 0.782577 | 0.092551 |
| Protein LZIC | Lzic | + | 1.965596 | **0.452565** | 9 | 1.617205 | 0.557493 | 0.73189 | 0.275763 |
| Transferrin receptor protein 1 | Tfrc | + | 3.269687 | **0.454438** | 27 | 2.10332 | 0.261135 | 0.955828 | 0.38851 |
| Fibulin-5 | Fbln5 | + | 2.521458 | **0.456053** | 8 | 0.282616 | 0.093796 | 0.128888 | 0.036176 |
| Neutral alpha-glucosidase C | Ganc | + | 2.540622 | **0.456448** | 13 | 0.200274 | 0.053906 | 0.091415 | 0.022333 |
| Transient receptor potential cation channel subfamily V member 2 | Trpv2 | + | 2.112869 | **0.458246** | 14 | 0.253358 | 0.052797 | 0.1161 | 0.067232 |
| Tropomodulin-3 | Tmod3 | + | 2.159103 | **0.460615** | 7 | 0.232861 | 0.089838 | 0.107259 | 0.025601 |
| Nuclear factor 1 A-type;  Nuclear factor 1 | Nfia | + | 3.635388 | **0.461598** | 8 | 1.19099 | 0.225274 | 0.549759 | 0.101236 |
| Integrin alpha-1 | Itga1 | + | 2.036165 | **0.463155** | 12 | 0.135468 | 0.011558 | 0.062743 | 0.036809 |
| Prefoldin subunit 3 | Vbp1 | + | 2.193151 | **0.463653** | 10 | 2.057811 | 0.535309 | 0.954111 | 0.361479 |
| TP53-regulating kinase | Tp53rk | + | 3.013757 | **0.46521** | 5 | 0.431174 | 0.089617 | 0.200587 | 0.063346 |
| Spermatid perinuclear RNA-binding protein | Strbp | + | 2.460396 | **0.466124** | 20 | 1.057455 | 0.351895 | 0.492905 | 0.071742 |
| Nuclear factor 1 B-type;Nuclear factor 1 | Nfib | + | 2.431228 | **0.468406** | 6 | 0.505968 | 0.101421 | 0.236998 | 0.090336 |
| DBIRD complex subunit ZNF326 | Znf326;  Zfp326 | + | 1.901663 | **0.469464** | 17 | 0.593418 | 0.106941 | 0.278588 | 0.108573 |
| Echinoderm microtubule-associated protein-like 4 | Eml4 | + | 3.103617 | **0.47076** | 22 | 0.452119 | 0.106493 | 0.21284 | 0.055382 |
| E3 ubiquitin-protein ligase NEDD4-like | Nedd4l | + | 4.124785 | **0.472687** | 34 | 1.018562 | 0.185388 | 0.481462 | 0.081005 |
| Rabenosyn-5 | Rbsn | + | 2.123661 | **0.473782** | 10 | 0.136848 | 0.036855 | 0.064836 | 0.02343 |
| Glutamine-rich protein 1 | Qrich1 | + | 2.683222 | **0.478657** | 9 | 0.318199 | 0.068702 | 0.152308 | 0.04607 |
| CSC1-like protein 2 | Tmem63b | + | 2.494355 | **0.479612** | 14 | 0.144661 | 0.022101 | 0.069381 | 0.028934 |
| cAMP-dependent protein kinase type II-beta regulatory subunit | Prkar2b | + | 2.607413 | **0.482093** | 23 | 2.293703 | 0.881666 | 1.105779 | 0.152932 |
| Lamin-B1 | Lmnb1 | + | 6.066767 | **0.482237** | 44 | 29.84608 | 2.919065 | 14.39288 | 1.037644 |
| Protein mab-21-like 1;Protein mab-21-like 2 | Mab21l1;  Mab21l2 | + | 2.191147 | **0.48368** | 6 | 0.62008 | 0.260403 | 0.29992 | 0.040264 |
| N(G),N(G)-dimethylarginine dimethylaminohydrolase 2 | Ddah2 | + | 4.138906 | **0.484339** | 16 | 8.957617 | 1.706977 | 4.338525 | 0.673046 |
|  | Acap3 | + | 2.454956 | **0.484688** | 15 | 0.130737 | 0.037584 | 0.063367 | 0.01948 |
| IgLON family member 5 | Iglon5 | + | 2.941271 | **0.485803** | 8 | 1.057778 | 0.204812 | 0.513872 | 0.146071 |
| Protein FAM171A2 | Fam171a2 | + | 3.072932 | **0.48804** | 20 | 0.65085 | 0.194942 | 0.317641 | 0.042954 |
| Catenin delta-1 | Ctnnd1 | + | 3.053215 | **0.48953** | 35 | 0.811661 | 0.107813 | 0.397332 | 0.120134 |
| Voltage-dependent R-type calcium channel subunit alpha-1E | Cacna1e | + | 2.012076 | **0.491163** | 47 | 0.116306 | 0.012019 | 0.057125 | 0.041936 |
| C-Jun-amino-terminal kinase-interacting protein 1 | Mapk8ip1 | + | 2.462309 | **0.493168** | 10 | 0.150981 | 0.047057 | 0.074459 | 0.011246 |
| Gelsolin | Gsn | + | 4.557244 | **0.495429** | 32 | 5.385898 | 0.577872 | 2.668329 | 0.431951 |
| Mitogen-activated protein kinase kinase kinase 7 | Map3k7 | + | 2.512094 | **0.496844** | 9 | 0.09522 | 0.015118 | 0.04731 | 0.015167 |
| Signal recognition particle 19 kDa protein | Srp19 | + | 3.599976 | **0.498499** | 6 | 1.986141 | 0.345941 | 0.99009 | 0.155656 |
| Laminin subunit beta-2 | Lamb2 | + | 4.672248 | **2.019463** | 60 | 0.641889 | 0.171765 | 1.296272 | 0.173096 |
| Brain-enriched guanylate kinase-associated protein | Begain | + | 2.109851 | **2.026053** | 21 | 0.114453 | 0.079399 | 0.231887 | 0.053629 |
| Septin-6 | 6-Sep | + | 4.37468 | **2.0275** | 17 | 2.911836 | 0.513387 | 5.903746 | 1.441293 |
| Semaphorin-7A | Sema7a | + | 3.322549 | **2.039823** | 20 | 0.536991 | 0.198949 | 1.095366 | 0.256589 |
| Transmembrane protein 177 | Tmem177 | + | 2.499473 | **2.041188** | 7 | 0.179374 | 0.089686 | 0.366136 | 0.043373 |
| Ubiquinol-cytochrome-c reductase complex assembly factor 1 | Uqcc1 | + | 3.08897 | **2.0459** | 7 | 0.376643 | 0.132563 | 0.770575 | 0.227512 |
| ADP-ribosylation factor-like protein 4C | Arl4c | + | 3.847598 | **2.049081** | 3 | 0.127461 | 0.069162 | 0.261178 | 0.044394 |
| Immunoglobulin superfamily member 8 | Igsf8 | + | 4.506395 | **2.050953** | 25 | 5.958436 | 1.044542 | 12.22048 | 2.317977 |
| Acyl-coenzyme A thioesterase THEM4 | Them4 | + | 3.911897 | **2.053671** | 8 | 1.558047 | 0.469363 | 3.199715 | 0.484025 |
| CTP synthase 2 | Ctps2 | + | 3.271955 | **2.056319** | 12 | 0.157118 | 0.049216 | 0.323085 | 0.05963 |
| Leucine-rich repeat LGI family member 2 | Lgi2 | + | 4.159973 | **2.056584** | 17 | 0.657065 | 0.161282 | 1.351309 | 0.286915 |
| Lamin-B2 | Lmnb2 | + | 6.586972 | **2.058178** | 37 | 5.800636 | 0.466936 | 11.93874 | 1.451521 |
| Alpha-2-macroglobulin;  Alpha-2-macroglobulin 165 kDa subunit;  Alpha-2-macroglobulin 35 kDa subunit | A2m;  Pzp | + | 4.853187 | **2.086928** | 45 | 0.887955 | 0.155464 | 1.853099 | 0.375833 |
| Phospholipid scramblase 3 | Plscr3 | + | 2.27654 | **2.09044** | 4 | 0.076427 | 0.04456 | 0.159766 | 0.054199 |
| Argininosuccinate synthase | Ass1;  Gm5424 | + | 4.391374 | **2.091336** | 24 | 0.769782 | 0.16519 | 1.609874 | 0.378624 |
| Kynurenine--oxoglutarate transaminase 3 | Ccbl2 | + | 4.566926 | **2.114036** | 12 | 0.377821 | 0.10837 | 0.798727 | 0.088508 |
| Hyaluronan and proteoglycan link protein 4 | Hapln4 | + | 4.898956 | **2.114834** | 21 | 1.880816 | 0.4063 | 3.977614 | 0.590099 |
| Serine/threonine-protein kinase STK11 | Stk11 | + | 2.558893 | **2.119831** | 8 | 0.045861 | 0.029033 | 0.097217 | 0.031102 |
| Aldehyde dehydrogenase family 3 member B1 | Aldh3b1 | + | 4.160629 | **2.127481** | 14 | 0.500354 | 0.127214 | 1.064493 | 0.179947 |
| Aspartoacylase | Aspa | + | 2.252305 | **2.14741** | 13 | 0.523227 | 0.350942 | 1.123582 | 0.123734 |
|  | Tex264 | + | 1.869059 | **2.150972** | 5 | 0.118696 | 0.063288 | 0.255313 | 0.097206 |
| Basal cell adhesion molecule | Bcam | + | 2.7535 | **2.151603** | 17 | 0.390433 | 0.231298 | 0.840056 | 0.08868 |
| Isobutyryl-CoA dehydrogenase, mitochondrial | Acad8 | + | 4.453888 | **2.188708** | 19 | 2.490502 | 0.309815 | 5.450982 | 1.635556 |
|  | Kcnab3 | + | 3.496677 | **2.202508** | 5 | 0.124659 | 0.038293 | 0.274562 | 0.078619 |
| FLYWCH-type zinc finger-containing protein 1 | Flywch1 | + | 2.046046 | **2.214289** | 6 | 0.055841 | 0.041731 | 0.123649 | 0.046669 |
| Guanine nucleotide-binding protein G(I)/G(S)/G(O) subunit gamma-10;Guanine nucleotide-binding protein subunit gamma | Gng10;  Gm20503 | + | 2.963007 | **2.257511** | 6 | 4.256586 | 1.628724 | 9.609291 | 1.038247 |
| 3-ketoacyl-CoA thiolase, mitochondrial | Acaa2 | + | 6.925624 | **2.262632** | 24 | 2.921099 | 0.500781 | 6.609372 | 0.624386 |
| Myelin-associated oligodendrocyte basic protein | Mobp | + | 5.565551 | **2.271596** | 19 | 17.04611 | 3.688914 | 38.72188 | 4.112373 |
| Lambda-crystallin homolog | Cryl1 | + | 4.264689 | **2.281029** | 15 | 0.64912 | 0.132123 | 1.480661 | 0.369801 |
| PDZ and LIM domain protein 1 | Pdlim1 | + | 2.434327 | **2.292169** | 7 | 0.140694 | 0.093997 | 0.322495 | 0.072205 |
| L-2-hydroxyglutarate dehydrogenase, mitochondrial | L2hgdh | + | 2.52014 | **2.317666** | 16 | 0.392787 | 0.169487 | 0.910349 | 0.356731 |
| SH3 domain-binding protein 1 | Sh3bp1 | + | 3.01426 | **2.32805** | 18 | 0.080952 | 0.024841 | 0.188459 | 0.078125 |
| Versican core protein | Vcan | + | 5.629355 | **2.373962** | 28 | 0.421018 | 0.090404 | 0.999482 | 0.132737 |
| NAD kinase 2, mitochondrial | Nadk2 | + | 5.654305 | **2.384792** | 19 | 1.26869 | 0.167766 | 3.025561 | 0.554211 |
| Complement C1q subcomponent subunit C | C1qc | + | 4.153837 | **2.398418** | 6 | 0.246883 | 0.134839 | 0.592128 | 0.099949 |
| Phosphatidylglycerophosphatase and protein-tyrosine phosphatase 1 | Ptpmt1 | + | 2.477267 | **2.435041** | 7 | 0.296234 | 0.110254 | 0.721342 | 0.349498 |
| Collagen alpha-1(VI) chain | Col6a1 | + | 3.644223 | **2.439898** | 16 | 0.070822 | 0.017834 | 0.172798 | 0.061249 |
| tRNA pseudouridine synthase A, mitochondrial;  tRNA pseudouridine synthase | Pus1 | + | 2.271732 | **2.448228** | 3 | 0.020215 | 0.016468 | 0.049491 | 0.031124 |
| Carbonic anhydrase 1 | Ca1 | + | 3.719952 | **2.457738** | 11 | 1.367815 | 0.485722 | 3.36173 | 0.868189 |
| Interferon-induced guanylate-binding protein 2 | Gbp2 | + | 3.8982 | **2.459904** | 7 | 0.05297 | 0.01603 | 0.1303 | 0.037817 |
| UPF0462 protein C4orf33 homolog | D3Ertd751e | + | 3.036975 | **2.472796** | 5 | 0.102199 | 0.063083 | 0.252718 | 0.096556 |
| Quinone oxidoreductase-like protein 2 | BC026585 | + | 3.184021 | **2.527566** | 12 | 0.432874 | 0.280226 | 1.094118 | 0.227702 |
| Pterin-4-alpha-carbinolamine dehydratase 2 | Pcbd2 | + | 2.268707 | **2.530602** | 3 | 0.299463 | 0.304628 | 0.757821 | 0.190115 |
| Mycophenolic acid acyl-glucuronide esterase, mitochondrial | Abhd10 | + | 2.790143 | **2.542629** | 15 | 0.602885 | 0.244066 | 1.532912 | 0.537088 |
| Pre-rRNA-processing protein TSR2 homolog | Tsr2 | + | 1.967101 | **2.570646** | 6 | 0.725054 | 0.614987 | 1.863857 | 0.752267 |
| Filamin-C | Flnc | + | 2.188222 | **2.580482** | 13 | 0.018959 | 0.008543 | 0.048924 | 0.029523 |
| Ubiquinone biosynthesis monooxygenase COQ6, mitochondrial | Coq6 | + | 1.998637 | **2.652472** | 13 | 0.295897 | 0.080536 | 0.784859 | 0.521822 |
| Palmitoyl-protein thioesterase 1 | Ppt1 | + | 5.059892 | **2.65461** | 8 | 0.876557 | 0.235636 | 2.326917 | 0.424068 |
| F-box/LRR-repeat protein 20 | Fbxl20 | + | 2.245122 | **2.692964** | 8 | 0.124847 | 0.112826 | 0.336208 | 0.062257 |
| Glutathione S-transferase theta-1 | Gstt1 | + | 3.726312 | **2.706424** | 6 | 0.213315 | 0.113663 | 0.57732 | 0.111106 |
| InaD-like protein | Inadl | + | 4.412818 | **2.73514** | 10 | 0.062784 | 0.019102 | 0.171723 | 0.045129 |
| Guanine nucleotide-binding protein G(I)/G(S)/G(O) subunit gamma-11 | Gng11 | + | 2.624515 | **2.787764** | 4 | 1.362217 | 0.818438 | 3.797541 | 1.075378 |
| Probable carboxypeptidase PM20D1 | Pm20d1 | + | 2.637577 | **2.839998** | 8 | 0.06174 | 0.052609 | 0.175341 | 0.070678 |
| 5-demethoxyubiquinone hydroxylase, mitochondrial | Coq7 | + | 2.556749 | **2.86538** | 6 | 0.904239 | 0.5248 | 2.590989 | 0.514421 |
| Cyclin-dependent kinase 14 | Cdk14 | + | 2.299395 | **2.893875** | 18 | 0.193524 | 0.056731 | 0.560034 | 0.486992 |
| Aggrecan core protein | Acan | + | 3.210407 | **2.908768** | 17 | 0.076168 | 0.049148 | 0.221555 | 0.035888 |
| UPF0515 protein C19orf66 homolog | | + | 2.07563 | **2.931952** | **6** | 0.110692 | 0.146903 | 0.324543 | 0.06272 |
| Ig mu chain C region | Ighm | + | 2.399592 | **3.207916** | 10 | 0.32606 | 0.605427 | 1.045972 | 0.460584 |
| O-acetyl-ADP-ribose deacetylase MACROD1 | Macrod1 | + | 4.894353 | **3.284187** | 7 | 0.621012 | 0.234968 | 2.039521 | 0.369634 |
| Protein THEM6 | Them6 | + | 2.122354 | **3.335555** | 14 | 0.824089 | 0.604531 | 2.748795 | 1.1004 |
| Ferritin heavy chain;Ferritin heavy chain, N-terminally processed | Fth1 | + | 5.515719 | **3.383011** | 13 | 7.972117 | 2.494325 | 26.96976 | 4.16945 |
| Ferritin light chain 1;Ferritin;Ferritin light chain 2 | Ftl1;Ftl2 | + | 5.442352 | **3.393381** | 10 | 2.49845 | 0.891316 | 8.478193 | 0.722121 |
| Ubiquinone biosynthesis O-methyltransferase, mitochondrial | Coq3 | + | 4.154444 | **3.424326** | 7 | 0.149755 | 0.057236 | 0.512808 | 0.184799 |
|  | Gbp6;  Gbp10 | + | 3.655079 | **3.457071** | 7 | 0.062483 | 0.042733 | 0.216007 | 0.074497 |
| Very long-chain acyl-CoA synthetase | Slc27a2 | + | 3.075587 | **3.54592** | 6 | 0.025852 | 0.01426 | 0.09167 | 0.032744 |
| 2-amino-3-ketobutyrate coenzyme A ligase, mitochondrial | Gcat | + | 2.277283 | **3.568965** | 9 | 0.283167 | 0.201082 | 1.010612 | 0.462692 |
| Ethanolamine-phosphate phospho-lyase | Etnppl | + | 2.455839 | **3.588201** | 8 | 0.19389 | 0.098968 | 0.695716 | 0.092903 |
| Inter-alpha-trypsin inhibitor heavy chain H3 | Itih3 | + | 4.956418 | **3.596743** | 10 | 0.051263 | 0.014503 | 0.184381 | 0.04672 |
| Tubulointerstitial nephritis antigen-like | Tinagl1 | + | 2.853989 | **3.666129** | 8 | 0.199567 | 0.104989 | 0.731639 | 0.141089 |
| Carbonic anhydrase 7 | Ca7;  Car7 | + | 3.437189 | **3.683764** | 6 | 0.379017 | 0.193073 | 1.39621 | 0.580228 |
| Leucine-rich repeat LGI family member 4 | Lgi4 | + | 4.448294 | **3.838567** | 13 | 0.290699 | 0.14193 | 1.115866 | 0.280827 |
| ATP-sensitive inward rectifier potassium channel 11 | Kcnj11 | + | 2.711254 | **4.056593** | 6 | 0.058964 | 0.077403 | 0.239191 | 0.098984 |
| H-2 class I histocompatibility antigen, K-D alpha chain | H2-K1 | + | 4.443121 | **4.078665** | 9 | 0.08891 | 0.03112 | 0.362632 | 0.126765 |
| Guanine nucleotide-binding protein G(I)/G(S)/G(O) subunit gamma-7;Guanine nucleotide-binding protein subunit gamma | Gng7 | + | 3.477512 | **4.285286** | 5 | 1.75887 | 0.777726 | 7.537259 | 4.426442 |
| Phospholysine phosphohistidine inorganic pyrophosphate phosphatase | Lhpp | + | 6.076266 | **4.796199** | 5 | 0.159506 | 0.090885 | 0.765024 | 0.136229 |
| Immunity-related GTPase family M protein 1 | Irgm1 | + | 3.727911 | **5.364792** | 10 | 0.085439 | 0.052704 | 0.45836 | 0.235844 |
| Hyaluronan and proteoglycan link protein 1 | Hapln1 | + | 5.810095 | **5.386061** | 24 | 1.45425 | 0.589529 | 7.832677 | 1.601196 |
| Optic atrophy 3 protein homolog | Opa3 | + | 2.814694 | **5.617787** | 7 | 0.245199 | 0.225591 | 1.377477 | 0.908873 |
| Isovaleryl-CoA dehydrogenase, mitochondrial | Ivd | + | 10.54604 | **5.970962** | 22 | 1.532283 | 0.196767 | 9.149201 | 0.727867 |
| Galectin-3-binding protein | Lgals3bp | + | 2.44534 | **6.017918** | 4 | 0.013338 | 0.016897 | 0.08027 | 0.024387 |
| Alpha-aminoadipic semialdehyde synthase, mitochondrial;  Lysine ketoglutarate reductase;  Saccharopine dehydrogenase | Aass | + | 5.104825 | **6.916585** | 12 | 0.037887 | 0.023196 | 0.262051 | 0.046802 |
| Cytochrome c oxidase subunit 7A-related protein, mitochondrial | Cox7a2l | + | 4.686408 | **6.997031** | 6 | 1.341106 | 0.552357 | 9.383757 | 4.789211 |
| Hyaluronan and proteoglycan link protein 2 | Hapln2 | + | 6.270571 | **12.08072** | 15 | 0.651475 | 0.26741 | 7.870289 | 2.243724 |
| Ig kappa chain C region | Igkc | + | 4.956123 | **12.72583** | 3 | 0.110618 | 0.141848 | 1.407701 | 0.390439 |
| Ig gamma-2B chain C region | Ighg2b;  Igh-3 | + | 6.239862 | **24.26264** | 12 | 0.070549 | 0.061354 | 1.711702 | 0.760041 |

**Part C. Cortex (pages 20-24)**

| **Protein**  **names** | **Gene**  **names** | **t-test Significant** | **-log(10)**  **of p-value** | **Ratio adult/young** | **Razor + unique peptides** | **Young** | | **Adult** | |
| --- | --- | --- | --- | --- | --- | --- | --- | --- | --- |
|  |  |  |  |  |  | **Mean concentration** | **SD** | **Mean concentration** | **SD** |
| Ceramide synthase 4 | Cers4 | + | 2.452537 | **0.119683** | 4 | 0.546698 | 0.462479 | 0.065431 | 0.08536 |
| Fatty acid-binding protein, brain | Fabp7 | + | 8.120433 | **0.121389** | 12 | 27.44714 | 3.637892 | 3.33178 | 0.83154 |
| 2-hydroxyacylsphingosine 1-beta-galactosyltransferase | Ugt8 | + | 3.65479 | **0.1243** | 17 | 0.534777 | 0.312589 | 0.066473 | 0.050172 |
| Poly(ADP-ribose) glycohydrolase | Parg | + | 3.556003 | **0.132973** | 5 | 0.047131 | 0.015706 | 0.006267 | 0.007097 |
| Dihydropyrimidinase-related protein 3 | Dpysl3 | + | 9.24311 | **0.159584** | 36 | 31.47856 | 3.470223 | 5.023489 | 0.814106 |
| MARCKS-related protein | Marcksl1 | + | 5.602125 | **0.159886** | 7 | 19.29639 | 4.301421 | 3.085221 | 1.05699 |
|  | Srcap | + | 2.995658 | **0.180625** | 7 | 0.061632 | 0.024014 | 0.011132 | 0.01779 |
| Mitochondrial 10-formyltetrahydrofolate dehydrogenase | Aldh1l2 | + | 3.166022 | **0.182696** | 17 | 0.200867 | 0.043066 | 0.036698 | 0.063795 |
| Coxsackievirus and adenovirus receptor homolog | Cxadr | + | 5.977233 | **0.204832** | 13 | 2.855281 | 0.825277 | 0.584854 | 0.092704 |
| Uncharacterized family 31 glucosidase KIAA1161 | Kiaa1161 | + | 3.642824 | **0.209345** | 10 | 0.064839 | 0.015373 | 0.013574 | 0.01157 |
| Lipid phosphate phosphatase-related protein type 3 | Lppr3 | + | 6.361893 | **0.217407** | 15 | 0.800367 | 0.189029 | 0.174005 | 0.04285 |
| Dephospho-CoA kinase domain-containing protein | Dcakd | + | 2.217021 | **0.245584** | 9 | 0.450786 | 0.230469 | 0.110706 | 0.092404 |
| Cadherin-6 | Cdh6 | + | 2.273702 | **0.259069** | 8 | 0.158607 | 0.056639 | 0.04109 | 0.036003 |
| Dihydropyrimidinase-related protein 5 | Dpysl5 | + | 5.68021 | **0.285619** | 39 | 22.18366 | 2.962997 | 6.336069 | 1.745766 |
| Serpin H1 | Serpinh1 | + | 4.416353 | **0.291859** | 10 | 0.480626 | 0.072003 | 0.140275 | 0.057183 |
| Prominin-1 | Prom1 | + | 3.705872 | **0.298369** | 14 | 0.242944 | 0.028923 | 0.072487 | 0.036145 |
| 39S ribosomal protein L15, mitochondrial | Mrpl15 | + | 2.53113 | **0.303567** | 14 | 0.594204 | 0.254073 | 0.18038 | 0.148994 |
| Tenascin | Tnc | + | 5.522699 | **0.304042** | 57 | 0.755683 | 0.153384 | 0.229759 | 0.05265 |
| NADH dehydrogenase [ubiquinone] complex I, assembly factor 7 | Ndufaf7 | + | 3.915147 | **0.305645** | 8 | 0.225562 | 0.070848 | 0.068942 | 0.020417 |
| Contactin-6 | Cntn6 | + | 2.915457 | **0.307345** | 7 | 0.06785 | 0.012276 | 0.020853 | 0.020001 |
| Transducin-like enhancer protein 3 | Tle3 | + | 2.772402 | **0.307655** | 6 | 0.069301 | 0.011872 | 0.021321 | 0.020407 |
| E3 ubiquitin-protein ligase RING1 | Ring1 | + | 2.499105 | **0.312298** | 3 | 0.181153 | 0.079497 | 0.056574 | 0.035354 |
| Neuron navigator 1 | Nav1 | + | 4.065161 | **0.341091** | 35 | 0.281054 | 0.033423 | 0.095865 | 0.037028 |
| D-beta-hydroxybutyrate dehydrogenase, mitochondrial | Bdh1 | + | 4.785117 | **0.349268** | 21 | 25.74609 | 5.191248 | 8.992285 | 1.949239 |
| Putative phospholipase B-like 2;  Putative phospholipase B-like 2 28 kDa form;  Putative phospholipase B-like 2 40 kDa form;  Putative phospholipase B-like 2 15 kDa form | Plbd2 | + | 2.826922 | **0.356013** | 10 | 0.201505 | 0.066216 | 0.071739 | 0.027572 |
| Methylmalonic aciduria type A homolog, mitochondrial | Mmaa | + | 4.03973 | **0.370175** | 12 | 0.477745 | 0.084043 | 0.176849 | 0.047702 |
| Beta-galactosidase | Glb1 | + | 2.797542 | **0.374858** | 11 | 0.328216 | 0.099658 | 0.123034 | 0.037788 |
|  | Kcnt2 | + | 2.98399 | **0.375933** | 7 | 0.055568 | 0.014709 | 0.02089 | 0.011682 |
| Neutral alpha-glucosidase C | Ganc | + | 3.528901 | **0.383745** | 13 | 0.125122 | 0.015615 | 0.048015 | 0.019735 |
| Fatty acyl-CoA reductase 1 | Far1 | + | 2.604053 | **0.386248** | 8 | 0.146137 | 0.021497 | 0.056445 | 0.04008 |
| Ubiquitin-conjugating enzyme E2 G1;  Ubiquitin-conjugating enzyme E2 G1, N-terminally processed | Ube2g1 | + | 2.697527 | **0.392082** | 5 | 0.454552 | 0.152409 | 0.178222 | 0.095652 |
| Ephrin type-B receptor 3 | Ephb3 | + | 2.498829 | **0.39669** | 17 | 0.220561 | 0.065026 | 0.087495 | 0.040735 |
| Mitogen-activated protein kinase-binding protein 1 | Mapkbp1 | + | 2.574479 | **0.396739** | 3 | 0.014148 | 0.00389 | 0.005613 | 0.003155 |
| Sentrin-specific protease 3 | Senp3 | + | 2.852384 | **0.397241** | 3 | 0.094093 | 0.02255 | 0.037378 | 0.029147 |
| Ras-related protein R-Ras2 | Rras2 | + | 4.214843 | **0.41743** | 12 | 2.708493 | 0.681654 | 1.130605 | 0.220616 |
|  | Scaper | + | 3.557874 | **0.419356** | 14 | 0.114557 | 0.037379 | 0.04804 | 0.004711 |
| Chondroitin sulfate proteoglycan 4 | Cspg4 | + | 3.260449 | **0.428126** | 42 | 0.276714 | 0.052513 | 0.118468 | 0.033916 |
| Rho GTPase-activating protein 33 | Arhgap33 | + | 2.425214 | **0.429855** | 17 | 0.243999 | 0.086548 | 0.104884 | 0.022758 |
| Receptor-type tyrosine-protein phosphatase F | Ptprf | + | 2.820969 | **0.433775** | 21 | 0.107985 | 0.030118 | 0.046841 | 0.008942 |
| Protein PRRC1 | Prrc1 | + | 3.604513 | **0.456059** | 8 | 0.450871 | 0.076406 | 0.205624 | 0.053447 |
| Transmembrane protein 214 | Tmem214 | + | 3.694902 | **0.457757** | 11 | 0.09993 | 0.026257 | 0.045743 | 0.005038 |
| Chondroadherin-like protein | Chadl | + | 2.472203 | **0.472877** | 10 | 0.10011 | 0.020522 | 0.04734 | 0.014494 |
| Nuclease-sensitive element-binding protein 1 | Ybx1 | + | 4.644344 | **0.473522** | 12 | 1.791285 | 0.312117 | 0.848213 | 0.137601 |
| 60S ribosomal protein L35a | Rpl35a | + | 2.726984 | **0.477408** | 14 | 15.88817 | 3.831396 | 7.585131 | 1.959556 |
| Alanine aminotransferase 2 | Gpt2 | + | 2.547622 | **0.477562** | 14 | 0.257348 | 0.040275 | 0.1229 | 0.047487 |
| Sphingosine kinase 2 | Sphk2 | + | 2.295924 | **0.480536** | 8 | 0.350325 | 0.093794 | 0.168344 | 0.069481 |
| Fatty acid-binding protein, epidermal | Fabp5 | + | 3.578502 | **0.480818** | 11 | 33.88201 | 8.661603 | 16.29106 | 2.564901 |
| Serine incorporator 1 | Serinc1 | + | 3.733204 | **0.483221** | 4 | 0.641919 | 0.150217 | 0.310189 | 0.042224 |
| Serine/threonine-protein kinase DCLK1 | Dclk1 | + | 5.407458 | **0.483729** | 42 | 12.43682 | 1.010816 | 6.016051 | 0.992579 |
| Rho guanine nucleotide exchange factor 2 | Arhgef2 | + | 6.3349 | **0.484119** | 51 | 3.272702 | 0.146504 | 1.584376 | 0.225904 |
|  | Fuk | + | 2.265021 | **2.001853** | 19 | 0.470599 | 0.169826 | 0.942071 | 0.268711 |
| Endophilin-A3 | Sh3gl3 | + | 3.927491 | **2.005924** | 17 | 1.743127 | 0.219822 | 3.496581 | 0.866261 |
| Methylcrotonoyl-CoA carboxylase subunit alpha, mitochondrial | Mccc1 | + | 4.066231 | **2.023775** | 24 | 0.526803 | 0.149991 | 1.066131 | 0.146881 |
| Neurofilament heavy polypeptide | Nefh | + | 3.501198 | **2.033783** | 46 | 3.469869 | 0.419325 | 7.056961 | 1.807123 |
| Serine beta-lactamase-like protein LACTB, mitochondrial | Lactb | + | 2.905543 | **2.038162** | 18 | 0.66908 | 0.252115 | 1.363694 | 0.270183 |
| Apoptosis-inducing factor 3 | Aifm3 | + | 2.96027 | **2.055234** | 15 | 0.418772 | 0.072731 | 0.860674 | 0.322654 |
| Coronin-6;  Coronin | Coro6 | + | 2.927517 | **2.103137** | 8 | 0.115052 | 0.038754 | 0.24197 | 0.072516 |
| Ribosyldihydronicotinamide dehydrogenase [quinone] | Nqo2 | + | 3.013216 | **2.124315** | 8 | 0.282178 | 0.095258 | 0.599435 | 0.139076 |
| Ubiquinone biosynthesis monooxygenase COQ6, mitochondrial | Coq6 | + | 2.825825 | **2.139107** | 13 | 0.229427 | 0.094779 | 0.490768 | 0.14553 |
|  | Mettl7a1;  Methig1;  UbiE2;  Mettl7a2 | + | 4.308586 | **2.143101** | 7 | 0.2986 | 0.075834 | 0.63993 | 0.144988 |
| Alpha-crystallin B chain | Cryab | + | 3.803139 | **2.156215** | 10 | 2.572267 | 0.561031 | 5.54636 | 1.596775 |
| Protein-arginine deiminase type-2 | Padi2 | + | 4.885364 | **2.157422** | 13 | 0.368448 | 0.077101 | 0.794897 | 0.152676 |
| Argininosuccinate synthase | Ass1;  Gm5424 | + | 5.209717 | **2.186894** | 24 | 2.301768 | 0.358914 | 5.033722 | 1.035983 |
| FAD-linked sulfhydryl oxidase ALR | Gfer | + | 4.01248 | **2.239042** | 6 | 0.608918 | 0.198066 | 1.363392 | 0.270085 |
| H-2 class I histocompatibility antigen, D-D alpha chain;  H-2 class I histocompatibility antigen, alpha chain;  H-2 class I histocompatibility antigen, D-P alpha chain | H2-D1 | + | 2.229519 | **2.275963** | 4 | 0.044842 | 0.023836 | 0.10206 | 0.071655 |
| Alpha-2-macroglobulin;  Alpha-2-macroglobulin 165 kDa subunit;Alpha-2-macroglobulin 35 kDa subunit | A2m;  Pzp | + | 3.674915 | **2.289834** | 45 | 0.554549 | 0.20726 | 1.269824 | 0.237574 |
| Major vault protein | Mvp | + | 2.906641 | **2.304748** | 20 | 0.099727 | 0.046108 | 0.229846 | 0.070244 |
| CD9 antigen | Cd9 | + | 3.546916 | **2.321964** | 5 | 2.546749 | 0.660461 | 5.91346 | 1.627232 |
| Immunoglobulin superfamily member 8 | Igsf8 | + | 3.642213 | **2.33962** | 25 | 4.299348 | 1.451456 | 10.05884 | 2.915322 |
| Palmitoyl-protein thioesterase 1 | Ppt1 | + | 4.388554 | **2.455878** | 8 | 0.846623 | 0.27057 | 2.079202 | 0.279812 |
| Unconventional myosin-Id | Myo1d | + | 3.720533 | **2.477024** | 47 | 0.316052 | 0.064893 | 0.782869 | 0.225796 |
| Serine protease HTRA1 | Htra1 | + | 2.358749 | **2.635861** | 11 | 0.177513 | 0.064535 | 0.467901 | 0.186853 |
| Calpastatin | Cast | + | 2.927846 | **2.652426** | 9 | 0.049804 | 0.03424 | 0.132101 | 0.036157 |
| Atypical kinase ADCK3, mitochondrial | Adck3 | + | 2.357627 | **2.713667** | 13 | 0.144266 | 0.092425 | 0.39149 | 0.203341 |
| Alpha-aminoadipic semialdehyde synthase, mitochondrial;  Lysine ketoglutarate reductase;  Saccharopine dehydrogenase | Aass | + | 3.062105 | **2.852758** | 12 | 0.053418 | 0.032925 | 0.152389 | 0.038116 |
| Prostaglandin G/H synthase 1 | Ptgs1 | + | 3.263337 | **2.881646** | 6 | 0.027038 | 0.023406 | 0.077913 | 0.04155 |
| Plasminogen;  Plasmin heavy chain A;  Activation peptide;  Angiostatin;  Plasmin heavy chain A, short form;  Plasmin light chain B | Plg | + | 2.432671 | **3.166273** | 9 | 0.031785 | 0.019641 | 0.10064 | 0.058969 |
| Very long-chain acyl-CoA synthetase | Slc27a2 | + | 3.19924 | **3.190101** | 6 | 0.017897 | 0.016699 | 0.057093 | 0.018379 |
| Myelin-associated oligodendrocyte basic protein | Mobp | + | 6.382017 | **3.209831** | 19 | 8.271789 | 1.754927 | 26.55105 | 5.782808 |
| Actin-binding protein anillin | Anln | + | 4.582867 | **3.345982** | 22 | 0.118874 | 0.037945 | 0.397749 | 0.094639 |
| Synaptophysin-like protein 1 | Sypl1;Sypl | + | 4.63559 | **3.355654** | 3 | 0.759482 | 0.156778 | 2.548559 | 0.985419 |
| Ubiquinone biosynthesis O-methyltransferase, mitochondrial | Coq3 | + | 4.488757 | **3.431263** | 7 | 0.138268 | 0.0474 | 0.474433 | 0.118461 |
| Tubulointerstitial nephritis antigen-like | Tinagl1 | + | 3.09579 | **3.587387** | 8 | 0.141007 | 0.053301 | 0.505847 | 0.26365 |
|  | Col4a5 | + | 2.683398 | **3.671227** | 3 | 0.013044 | 0.014351 | 0.047886 | 0.030784 |
| Leukocyte elastase inhibitor A | Serpinb1a | + | 7.384715 | **3.805763** | 19 | 0.542986 | 0.098788 | 2.066475 | 0.400106 |
| Aldehyde dehydrogenase family 3 member B1 | Aldh3b1 | + | 6.880112 | **4.122308** | 14 | 0.207965 | 0.03473 | 0.857295 | 0.212397 |
| Hyaluronan and proteoglycan link protein 2 | Hapln2 | + | 4.495006 | **4.313513** | 15 | 0.488991 | 0.10665 | 2.109268 | 0.823813 |
| Hydroxyacid-oxoacid transhydrogenase, mitochondrial | Adhfe1 | + | 3.51433 | **4.514825** | 7 | 0.024733 | 0.031489 | 0.111664 | 0.032858 |
| Carbonic anhydrase 1 | Ca1 | + | 4.201343 | **4.828228** | 11 | 0.453545 | 0.167568 | 2.189819 | 1.198774 |
| Guanine nucleotide-binding protein G(I)/G(S)/G(O) subunit gamma-11 | Gng11 | + | 3.438447 | **4.918654** | 4 | 0.192561 | 0.134054 | 0.947139 | 0.353902 |
| Retinol dehydrogenase 13 | Rdh13 | + | 3.591687 | **5.010784** | 6 | 0.056354 | 0.050059 | 0.28238 | 0.103765 |
| Isovaleryl-CoA dehydrogenase, mitochondrial | Ivd | + | 9.462436 | **5.170722** | 22 | 1.202942 | 0.203982 | 6.220081 | 0.72789 |
| SWI/SNF-related matrix-associated actin-dependent regulator of chromatin subfamily A-like protein 1 | Smarcal1 | + | 2.381999 | **6.384873** | 14 | 0.059796 | 0.033828 | 0.381792 | 0.330341 |
| Prosaposin receptor GPR37 | Gpr37 | + | 3.603852 | **7.235205** | 5 | 0.027763 | 0.020248 | 0.200868 | 0.07301 |
| Ig gamma-2B chain C region | Ighg2b;  Igh-3 | + | 6.697202 | **8.788042** | 12 | 0.181309 | 0.03725 | 1.593351 | 0.625193 |
| Interferon-induced guanylate-binding protein 2 | Gbp2 | + | 2.770103 | **8.818062** | 7 | 0.011848 | 0.013152 | 0.10448 | 0.077207 |
| Cytochrome c oxidase subunit 7A-related protein, mitochondrial | Cox7a2l | + | 3.542673 | **11.65296** | 6 | 0.811188 | 0.553943 | 9.452749 | 5.335695 |
