# Supplementary material for: Global quantitative TPA-based proteomics of mouse brain structures reveals significant alterations in expression of proteins involved in neuronal plasticity during aging
Source: Aging (Albany NY). 2018 Jul 19;10(7):1682–97. doi: 10.18632/aging.101501 (PMC6075443; doi:10.18632/aging.101501)
Supplement: Supplementary Table S2 [file aging-10-101501-s002.docx]

**Supplementary Table S2. Concentration of protein families and the statistical significance of their structure-to-structure variation.**

| **Protein family** | **Brain structure** | **Young** | | **Old** | | **Ratio Old / Young** | **T-test Significant** | **Significance of the structure-to-structure variation** | | | | |
| --- | --- | --- | --- | --- | --- | --- | --- | --- | --- | --- | --- | --- |
|  |  | **Average** | **SD** | **Average** | **SD** |  |  | **Protein family** | **Young**  (T-test Significant) | | **Old**  (T-test Significant) | |
| **Gria** | **Hippocampus** | 16.84 | 1.31 | 16.08 | 0.8683 | 0.9549 | 0.3097 | **Gria** | **Hip/Cer** | 1.93E-05 | **Hip/Cer** | 1.82E-07 |
|  | **Cerebellum** | 9.746 | 1.58 | 8.428 | 0.4747 | 0.8647 | 0.1251 |  | **Hip/Cor** | 1.69E-05 | **Hip/Cor** | 7.31E-07 |
|  | **Cortex** | 9.881 | 0.5351 | 10.31 | 0.6036 | 1.044 | 0.2538 |  | **Cer/Cor** | 0.8624 | **Cer/Cor** | 0.000316 |
| **SynDIG4**  **/Prrt1** | **Hippocampus** | 3.079 | 0.5311 | 2.175 | 0.2524 | 0.7063 | 0.01051 | **SynDIG4/Prrt1** | **Hip/Cer** |  | **Hip/Cer** |  |
|  | **Cerebellum** |  |  |  |  |  |  |  | **Hip/Cor** | 0.00022 | **Hip/Cor** | 1.73E-05 |
|  | **Cortex** | 1.186 | 0.2013 | 1.009 | 0.2234 | 0.8513 | 0.2196 |  | **Cer/Cor** |  | **Cer/Cor** |  |
| **Grik** | **Hippocampus** | 0.3029 | 0.07064 | 0.1975 | 0.01903 | 0.6521 | 0.003342 | **Grik** | **Hip/Cer** | 0.4733 | **Hip/Cer** | 0.000907 |
|  | **Cerebellum** | 0.3047 | 0.0652 | 0.2877 | 0.04214 | 0.9442 | 0.6368 |  | **Hip/Cor** | 0.000461 | **Hip/Cor** | 0.002974 |
|  | **Cortex** | 0.5 | 0.07617 | 0.3414 | 0.07431 | 0.6828 | 0.007593 |  | **Cer/Cor** | 0.001516 | **Cer/Cor** | 0.1982 |
| **Grin** | **Hippocampus** | 6.193 | 2.584 | 6.317 | 0.3309 | 1.02 | 0.5619 | **Grin** | **Hip/Cer** | 2.01E-09 | **Hip/Cer** | 1.21E-08 |
|  | **Cerebellum** | 1.005 | 0.1749 | 0.9445 | 0.1349 | 0.9395 | 0.5533 |  | **Hip/Cor** | 3.27E-05 | **Hip/Cor** | 1.89E-08 |
|  | **Cortex** | 3.771 | 0.5625 | 2.731 | 0.3677 | 0.7242 | 0.007656 |  | **Cer/Cor** | 4.59E-05 | **Cer/Cor** | 3.69E-05 |
| **Grid** | **Hippocampus** | 0.3623 | 0.04932 | 0.3344 | 0.1002 | 0.9229 | 0.5929 | **Grid** | **Hip/Cer** | 8.30E-06 | **Hip/Cer** | 8.48E-07 |
|  | **Cerebellum** | 6.687 | 0.7703 | 5.768 | 0.4803 | 0.8624 | 0.05179 |  | **Hip/Cor** | 0.000675 | **Hip/Cor** | 0.01661 |
|  | **Cortex** | 0.2178 | 0.04435 | 0.1802 | 0.04342 | 0.8274 | 0.2054 |  | **Cer/Cor** | 7.50E-06 | **Cer/Cor** | 1.35E-06 |
| **Grm** | **Hippocampus** | 6.087 | 0.5588 | 5.464 | 0.5898 | 0.8975 | 0.1169 | **Grm** | **Hip/Cer** | 0.09214 | **Hip/Cer** | 0.1966 |
|  | **Cerebellum** | 5.251 | 0.8176 | 4.941 | 0.6042 | 0.941 | 0.5128 |  | **Hip/Cor** | 0.579 | **Hip/Cor** | 0.4182 |
|  | **Cortex** | 6.304 | 0.631 | 5.775 | 0.5749 | 0.9161 | 0.1963 |  | **Cer/Cor** | 0.04744 | **Cer/Cor** | 0.04953 |
| **Gabbr** | **Hippocampus** | 5.093 | 0.6023 | 4.491 | 0.2086 | 0.8817 | 0.07763 | **Gabbr** | **Hip/Cer** | 0.003696 | **Hip/Cer** | 5.57E-05 |
|  | **Cerebellum** | 3.835 | 0.3373 | 3.238 | 0.3197 | 0.8443 | 0.01667 |  | **Hip/Cor** | 0.08946 | **Hip/Cor** | 0.004992 |
|  | **Cortex** | 4.378 | 0.6009 | 3.56 | 0.4692 | 0.8131 | 0.03872 |  | **Cer/Cor** | 0.1168 | **Cer/Cor** | 0.2378 |
| **Gabra** | **Hippocampus** | 4.53 | 0.8308 | 3.393 | 0.52 | 0.749 | 0.03071 | **Gabra** | **Hip/Cer** | 0.141 | **Hip/Cer** | 0.002764 |
|  | **Cerebellum** | 5.328 | 0.745 | 6.253 | 1.277 | 1.173 | 0.1994 |  | **Hip/Cor** | 0.8366 | **Hip/Cor** | 0.7364 |
|  | **Cortex** | 4.676 | 1.291 | 3.539 | 0.7835 | 0.7569 | 0.1298 |  | **Cer/Cor** | 0.3564 | **Cer/Cor** | 0.003423 |
| **Gad** | **Hippocampus** | 5.298 | 0.7913 | 6.158 | 0.4807 | 1.162 | 0.05151 | **Gad** | **Hip/Cer** | 0.1745 | **Hip/Cer** | 0.002458 |
|  | **Cerebellum** | 4.697 | 0.5621 | 5.148 | 0.3299 | 1.096 | 0.1598 |  | **Hip/Cor** | 0.1801 | **Hip/Cor** | 0.07664 |
|  | **Cortex** | 4.679 | 0.6315 | 5.418 | 0.6933 | 1.158 | 0.1086 |  | **Cer/Cor** | 0.9625 | **Cer/Cor** | 0.4568 |
| **Camk2** | **Hippocampus** | 137.1 | 5.844 | 127.5 | 3.002 | 0.9302 | 0.00824 | **Camk2** | **Hip/Cer** | 2.96E-09 | **Hip/Cer** | 3.76E-12 |
|  | **Cerebellum** | 44.86 | 2.537 | 42 | 1.658 | 0.9361 | 0.02899 |  | **Hip/Cor** | 0.1278 | **Hip/Cor** | 0.178073 |
|  | **Cortex** | 130.9 | 6.311 | 137.7 | 14.44 | 1.051 | 0.3688 |  | **Cer/Cor** | 4.06E-08 | **Cer/Cor** | 2.15E-05 |
| **Camk4** | **Hippocampus** | 2.812 | 0.478 | 1.77 | 0.0732 | 0.6296 | 0.002841 | **Camk4** | **Hip/Cer** | 3.71E-06 | **Hip/Cer** | 1.30E-06 |
|  | **Cerebellum** | 13.75 | 1.429 | 9.392 | 0.6549 | 0.6828 | 8.36E-06 |  | **Hip/Cor** | 0.004367 | **Hip/Cor** | 0.000856 |
|  | **Cortex** | 4.133 | 0.6425 | 2.527 | 0.2561 | 0.6114 | 0.001554 |  | **Cer/Cor** | 2.76E-06 | **Cer/Cor** | 2.54E-07 |
| **Prka** | **Hippocampus** | 28.17 | 1.881 | 28.77 | 0.5378 | 1.021 | 0.6885 | **Prka** | **Hip/Cer** | 4.61E-06 | **Hip/Cer** | 1.62E-07 |
|  | **Cerebellum** | 17.63 | 1.866 | 16.03 | 1.225 | 0.9093 | 0.1455 |  | **Hip/Cor** | 0.002061 | **Hip/Cor** | 0.14976 |
|  | **Cortex** | 38.83 | 4.517 | 30.44 | 2.168 | 0.7838 | 0.00687 |  | **Cer/Cor** | 3.61E-05 | **Cer/Cor** | 1.36E-06 |
| **Mapk** | **Hippocampus** | 29.95 | 2.009 | 35.22 | 2.852 | 1.175 | 0.008194 | **Mapk** | **Hip/Cer** | 1.13E-06 | **Hip/Cer** | 3.76E-06 |
|  | **Cerebellum** | 13.56 | 0.811 | 11 | 0.6221 | 0.8112 | 0.000288 |  | **Hip/Cor** | 4.38E-06 | **Hip/Cor** | 8.79E-06 |
|  | **Cortex** | 18.71 | 1.962 | 20.64 | 2.709 | 1.103 | 0.229 |  | **Cer/Cor** | 0.001165 | **Cer/Cor** | 0.00036 |

Hip – hippocampus

Cer – cerebellum

Cor - cortex
