# Supplementary material for: Global quantitative TPA-based proteomics of mouse brain structures reveals significant alterations in expression of proteins involved in neuronal plasticity during aging
Source: Aging (Albany NY). 2018 Jul 19;10(7):1682–97. doi: 10.18632/aging.101501 (PMC6075443; doi:10.18632/aging.101501)
Supplement: Supplementary Table S3 [file aging-10-101501-s003.docx]

**Supplementary Table S3. Concentrations of identified proteins involved in glutamate and GABA plasticity.**

|  |  | **Hippocampus** | | | | | | **Cerebellum** | | | | | | **Cortex** | | | | | |
| --- | --- | --- | --- | --- | --- | --- | --- | --- | --- | --- | --- | --- | --- | --- | --- | --- | --- | --- | --- |
|  |  | **Old / Young Ratio** | **T-test significant** | **Average Young** | **SD Young** | **Average Old** | **SD Old** | **Old / Young Ratio** | **T-test significant** | **Average Young** | **SD Young** | **Average Old** | **SD Old** | **Old / Young Ratio** | **T-test significant** | **Average Young** | **SD Young** | **Average Old** | **SD Old** |
| **Glutamatergic transmission** | **Gria1** | 0.8247 | 0.0202 | 4.4907 | 0.5317 | 3.703 | 0.1225 | 0.8888 | 0.2729 | 2.551 | 0.4280 | 2.267 | 0.3365 | 1.028 | 0.8412 | 1.464 | 0.1414 | 1.505 | 0.4161 |
|  | **Gria2** | 1.021 | 0.6665 | 10.59 | 0.9054 | 10.82 | 0.7200 | 0.8844 | 0.4854 | 4.080 | 1.365 | 3.608 | 0.3662 | 1.047 | 0.2700 | 7.219 | 0.4004 | 7.563 | 0.5207 |
|  | **Gria3** | 0.8145 | 0.0769 | 1.105 | 0.2037 | 0.9003 | 0.0759 | 1.093 | 0.5426 | 0.5009 | 0.1325 | 0.5477 | 0.0990 | 0.8849 | 0.1737 | 0.8369 | 0.1064 | 0.7407 | 0.1013 |
|  | **Gria4** | 1.006 | 0.9280 | 0.6498 | 0.0749 | 0.6538 | 0.0592 | 0.7665 | 0.000202 | 2.614 | 0.1726 | 2.004 | 0.1665 | 1.410 | 0.1515 | 0.3602 | 0.1485 | 0.5083 | 0.1529 |
|  | **SynDIG4/Prrt1** |  |  |  |  |  |  |  |  |  |  |  |  |  |  |  |  |  |  |
|  |  | 0.7063 | 0.01051 | 3.079 | 0.5311 | 2.175 | 0.2524 |  |  |  |  |  |  | 0.8513 | 0.2196 | 1.186 | 0.2013 | 1.009 | 0.2234 |
|  |  |  |  |  |  |  |  |  |  |  |  |  |  |  |  |  |  |  |  |
|  | **Grik2** | 0.5421 | 0.001124 | 0.1994 | 0.0328 | 0.1081 | 0.0090 | 0.9442 | 0.6368 | 0.3047 | 0.0652 | 0.2877 | 0.0421 | 0.7125 | 0.08101 | 0.2943 | 0.0691 | 0.2097 | 0.0687 |
|  | **Grik3** | 0.9268 | 0.1126 | 0.05311 | 0.0344 | 0.0492 | 0.0090 |  |  |  |  |  |  | 0.4934 | 0.07134 | 0.1464 | 0.0697 | 0.0722 | 0.0381 |
|  | **Grik5** |  |  |  |  |  |  |  |  |  |  |  |  | 1.003 | 0.9901 | 0.05920 | 0.0195 | 0.0593 | 0.0281 |
|  | **Grin1** | 1.023 | 0.6680 | 4.020 | 1.726 | 4.115 | 0.3531 | 1.031 | 0.8301 | 0.6460 | 0.1684 | 0.6665 | 0.1214 | 0.6988 | 0.01174 | 2.514 | 0.4513 | 1.757 | 0.2608 |
|  | **Grin2a** | 1.220 | 0.01141 | 0.6005 | 0.4970 | 0.7331 | 0.0483 | 1.135 | 0.5334 | 0.04771 | 0.0138 | 0.0541 | 0.0175 | 0.9076 | 0.4579 | 0.2393 | 0.0447 | 0.2172 | 0.0457 |
|  | **Grin2b** | 0.9341 | 0.1388 | 1.572 | 0.1258 | 1.468 | 0.0590 | 0.4780 | 0.0476 | 0.07309 | 0.0298 | 0.0349 | 0.0224 | 0.7341 | 0.01260 | 1.008 | 0.1598 | 0.7407 | 0.1046 |
|  | **Grin2c** |  |  |  |  |  |  | 0.7921 | 0.1596 | 0.2384 | 0.0581 | 0.1888 | 0.0431 | 1.814 | 0.2747 | 0.00917 | 0.0103 | 0.0166 | 0.0100 |
|  | **Grid1** | 0.9123 | 0.6459 | 0.2583 | 0.0531 | 0.2356 | 0.0917 | 0.5572 | 0.3022 | 0.04220 | 0.0355 | 0.0235 | 0.0096 | 0.8328 | 0.323 | 0.08101 | 0.0203 | 0.0674 | 0.020929 |
|  | **Grid2** | 0.9492 | 0.7654 | 0.1039 | 0.0292 | 0.0987 | 0.0250 | 0.8643 | 0.05275 | 6.645 | 0.7571 | 5.7445 | 0.4768 | 0.8242 | 0.3944 | 0.1368 | 0.0321 | 0.1128 | 0.050584 |
|  | **Grm1** | 0.9719 | 0.8174 | 0.3552 | 0.0342 | 0.3452 | 0.0861 | 0.9505 | 0.6498 | 2.731 | 0.6156 | 2.596 | 0.1284 | 1.092 | 0.7649 | 0.2423 | 0.0962 | 0.2647 | 0.1308 |
|  | **Grm2** | 0.8933 | 0.4015 | 1.397 | 0.3492 | 1.248 | 0.1231 | 1.343 | 0.1848 | 0.7318 | 0.1166 | 0.9835 | 0.3576 | 1.036 | 0.8194 | 1.758 | 0.3920 | 1.823 | 0.4766 |
|  | **Grm3** | 0.9497 | 0.4014 | 1.654 | 0.1503 | 1.571 | 0.1499 | 1.133 | 0.3370 | 0.5748 | 0.1136 | 0.6515 | 0.1265 | 0.8671 | 0.1383 | 2.566 | 0.3189 | 2.225 | 0.3494 |
|  | **Grm4** | 0.8685 | 0.7147 | 0.04238 | 0.0299 | 0.0368 | 0.0130 | 0.5510 | 0.000279 | 0.9995 | 0.1089 | 0.5507 | 0.1414 | 0.9270 | 0.6930 | 0.1112 | 0.0336 | 0.1031 | 0.0292 |
|  | **Grm5** | 0.8393 | 0.07706 | 2.283 | 0.2206 | 1.916 | 0.3434 | 0.7191 | 0.006465 | 0.1828 | 0.0211 | 0.1314 | 0.0256 | 0.8922 | 0.3574 | 1.185 | 0.4709 | 1.057 | 0.2375 |
|  | **Grm7** | 0.9752 | 0.8038 | 0.3544 | 0.0675 | 0.3456 | 0.0352 | 0.9102 | 0.8348 | 0.03081 | 0.0153 | 0.0280 | 0.0242 | 0.5240 | 0.000234 | 0.3523 | 0.0504 | 0.1846 | 0.0431 |
|  | **Grm8** |  |  |  |  |  |  |  |  |  |  |  |  | 1.324 | 0.2948 | 0.08813 | 0.0343 | 0.1167 | 0.0461 |
| **GABAergic transmission** | **Gabbr1** | 0.9545 | 0.6002 | 1.192 | 0.2073 | 1.138 | 0.0722 | 0.8462 | 0.1518 | 1.103 | 0.1054 | 0.9341 | 0.2125 | 0.6812 | 0.02652 | 1.684 | 0.3650 | 1.147 | 0.2728 |
|  | **Gabbr2** | 0.9519 | 0.6300 | 1.906 | 0.3894 | 1.8145 | 0.1027 | 0.8436 | 0.03612 | 2.731 | 0.3163 | 2.304 | 0.2254 | 0.8956 | 0.1261 | 2.693 | 0.2904 | 2.412 | 0.2377 |
|  | **Gabbr3** | 0.7711 | 0.01153 | 1.995 | 0.2730 | 1.538 | 0.1418 |  |  |  |  |  |  |  |  |  |  |  |  |
|  | **Gabra1** | 0.6959 | 0.03390 | 3.268 | 0.7527 | 2.274 | 0.4365 | 1.565 | 0.09458 | 2.008 | 0.5868 | 3.143 | 1.185 | 0.6942 | 0.08264 | 3.794 | 1.148 | 2.634 | 0.6050 |
|  | **Gabra2** | 0.9518 | 0.7672 | 0.7834 | 0.2159 | 0.7456 | 0.1737 | 0.7920 | 0.6318 | 0.1645 | 0.0778 | 0.1303 | 0.1325 | 1.284 | 0.3243 | 0.2421 | 0.1089 | 0.3112 | 0.1013 |
|  | **Gabra3** | 1.226 | 0.5512 | 0.08657 | 0.0539 | 0.1061 | 0.0462 |  |  |  |  |  |  | 0.8214 | 0.2474 | 0.4042 | 0.0914 | 0.3320 | 0.0944 |
|  | **Gabra4** | 1.239 | 0.6911 | 0.04563 | 0.0550 | 0.0565 | 0.0207 |  |  |  |  |  |  | 0.7934 | 0.03593 | 0.1965 | 0.0295 | 0.1559 | 0.0223 |
|  | **Gabra5** | 0.6079 | 0.04748 | 0.3461 | 0.1164 | 0.2104 | 0.0243 |  |  |  |  |  |  | 2.731 | 0.02353 | 0.03893 | 0.0418 | 0.1063 | 0.0378 |
|  | **Gabra6** |  |  |  |  |  |  | 0.9440 | 0.3900 | 3.156 | 0.2849 | 2.979 | 0.3341 |  |  |  |  |  |  |
|  | **Gad1** | 1.206 | 0.02255 | 2.351 | 0.3398 | 2.836 | 0.2758 | 1.074 | 0.3303 | 3.199 | 0.4840 | 3.437 | 0.1176 | 1.248 | 0.1791 | 2.341 | 0.5422 | 2.9229 | 0.7120 |
|  | **Gad2** | 1.127 | 0.1260 | 2.946 | 0.4647 | 3.321 | 0.2713 | 1.142 | 0.1517 | 1.497 | 0.1175 | 1.711 | 0.2717 | 1.067 | 0.5219 | 2.337 | 0.4152 | 2.495 | 0.3317 |
| **Kinases** | **Camk2a** | 0.9098 | 0.004105 | 114.1 | 5.460 | 103.8 | 2.355 | 0.9965 | 0.9481 | 20.69 | 2.145 | 20.62 | 0.9373 | 1.057 | 0.4412 | 102.0 | 7.132 | 107.8 | 14.32 |
|  | **Camk2b** | 1.015 | 0.8512 | 18.93 | 3.508 | 19.22 | 0.5603 | 0.9209 | 0.1919 | 16.51 | 1.804 | 15.21 | 0.9446 | 0.9384 | 0.06157 | 18.45 | 1.058 | 17.31 | 0.2626 |
|  | **Camk2g** | 1.084 | 0.1791 | 1.399 | 0.1654 | 1.517 | 0.1089 | 1.408 | 0.01450 | 2.362 | 0.6068 | 3.326 | 0.2958 | 1.204 | 0.1656 | 5.387 | 0.4011 | 6.487 | 1.494 |
|  | **Camk2d** | 1.123 | 0.6297 | 2.655 | 0.9645 | 2.983 | 1.293 | 0.8356 | 0.1485 | 7.565 | 1.379 | 6.321 | 1.107 | 1.194 | 0.2674 | 5.108 | 1.008 | 6.100 | 1.574 |
|  | **Camk4** | 0.6296 | 0.002841 | 2.812 | 0.4780 | 1.770 | 0.0732 | 0.6828 | 0.000441 | 13.75 | 1.429 | 9.392 | 0.6549 | 0.6114 | 0.001554 | 4.133 | 0.6425 | 2.527 | 0.2561 |
|  | **Prkar1a** | 0.9504 | 0.09864 | 2.021 | 0.1847 | 1.921 | 0.2115 | 0.9768 | 0.8199 | 3.763 | 0.5985 | 3.676 | 0.5824 | 0.8628 | 0.2215 | 3.208 | 0.5070 | 2.768 | 0.5581 |
|  | **Prkar1b** | 0.9398 | 0.007134 | 2.022 | 0.1702 | 1.900 | 0.1369 | 0.9544 | 0.4581 | 1.989 | 0.1753 | 1.899 | 0.1954 | 0.8679 | 0.1312 | 2.736 | 0.4158 | 2.375 | 0.2408 |
|  | **Prkar2a** | 1.206 | 0.02468 | 6.126 | 0.6228 | 7.393 | 0.3590 | 0.9291 | 0.6468 | 2.013 | 0.6108 | 1.870 | 0.2644 | 1.327 | 0.1844 | 3.051 | 0.7199 | 4.051 | 1.353 |
|  | **Prkar2b** | 0.8199 | 0.08290 | 7.495 | 1.060 | 6.145 | 0.3111 | 0.4820 | 0.02081 | 2.293 | 0.8048 | 1.105 | 0.1396 | 0.6109 | 0.00147 | 14.19 | 2.127 | 8.669 | 0.6793 |
|  | **Prkaca** | 1.179 | 0.8786 | 2.847 | 0.2959 | 3.359 | 0.3173 | 1.011 | 0.9073 | 6.942 | 0.9622 | 7.023 | 1.152 | 0.7937 | 0.05527 | 14.68 | 2.534 | 11.65 | 1.729 |
|  | **Prkacb** | 1.051 | 0.8322 | 7.660 | 0.8672 | 8.056 | 0.5172 | 0.7683 | 0.09285 | 0.5120 | 0.0906 | 0.3934 | 0.1096 | 0.9546 | 0.7395 | 0.9687 | 0.1907 | 0.9247 | 0.2151 |
|  | **Mapk1** | 1.221 | 0.00234 | 24.69 | 1.018 | 30.17 | 2.382 | 0.7551 | 0.003396 | 12.91 | 1.479 | 9.760 | 0.6865 | 1.103 | 0.2187 | 16.66 | 1.720 | 18.39 | 2.376 |
|  | **Mapk3** | 0.9603 | 0.7266 | 5.258 | 1.042 | 5.049 | 0.7675 | 1.074 | 0.5915 | 1.671 | 0.3946 | 1.795 | 0.3067 | 1.095 | 0.5138 | 2.049 | 0.5150 | 2.246 | 0.3912 |
